# In-silico study of drug delivery to atherosclerosis in the human carotid artery using metal–organic frameworks based on adhesion of nanocarriers

**DOI:** 10.1038/s41598-023-48803-0

**Published:** 2023-12-06

**Authors:** Amir Shamloo, Tahoora Naseri, Ali Rahbary, Mohammad Ali Bakhtiari, Sina Ebrahimi, Iman Mirafzal

**Affiliations:** 1 School of Mechanical Engineering, Sharif University of Technology, Azadi Ave., Tehran, Iran; 2 Stem Cell and Regenerative Medicine Institute, Sharif University of Technology, Tehran, Iran

**Keywords:** Computational biophysics, Molecular biophysics

## Abstract

This study investigates nanocarriers (NCs) for drug delivery targeting carotid artery atherosclerosis. This targeted drug delivery mechanism is based on ligand–receptor bindings facilitated by coating NCs with P-selectin aptamers, which exhibit high affinities for P-selectin plaque receptors. Recognizing the significant advantages of metal–organic frameworks (MOFs), such as their high drug-loading percentages, we chose them as nanocarriers for this research. Our evaluation considers critical factors: NC surface density (the number of attached nanocarriers per unit of plaque area), toxicity (percentage of NCs missing the target), and efficient drug transfer to plaque tissue. Employing molecular dynamics (MD) for drug loading calculations via van der Waals interactions and computational fluid dynamics (CFD) for toxicity, surface density, and drug transfer assessments, we achieve a comprehensive analysis. A cardiac cycle-based metric guides optimal MOF release conditions, establishing an ideal dosage of 600 NCs per cycle. MOF-801 exhibits outstanding drug delivery performance, particularly in plaque targeting. While a magnetic field enhances NC adhesion, its impact on drug transfer is limited, emphasizing the need for further optimization in magnetic targeting for NC-based therapies. This study provides crucial insights into NC drug delivery performance in carotid artery atherosclerosis, advancing the field of targeted drug delivery for atherosclerosis treatment.

## Introduction

Atherosclerosis is one of the most crucial factors in vascular diseases that cause heart attacks and strokes^1^. Improper blood cholesterol levels, which result in a decrease in high-density lipoprotein and an increase in low-density lipoprotein, lead to lipid plaque production with large necrotic nuclei and thin fibrous caps in the walls of blood vessels. These plaques are prone to rupture and can injure the inner layers of the artery walls. In this situation, the accumulation of platelets causes blood clots, arterial lumen narrowing, artery occlusion, and even death^2^. According to reports, about 7.6 million Americans over the age of 20 have suffered a stroke, and it is predicted that more than 3.4 million American adults will experience a stroke by 2030^3^. Since atherosclerosis is a complex disease with slow progress and the symptoms can be identified after the disease develops, a CT scan, MRI, and cardiac and brain angiography should be taken for diagnosis.

Furthermore, one conventional treatment method is stents and balloon angioplasty, or coronary artery transplantation^4,5^. With the development of nanotechnology in the future, it will be possible to replace invasive surgical methods with advanced molecular imaging technology to identify atherosclerotic plaques and evaluate the effectiveness of drugs and treatments. In addition, carrying nanoparticles to atherosclerosis plaques helps suppress the process of macrophages and healing the inflammation of the artery walls and accelerates the treatment of the disease^2,6^.

Continuous nanoscale particle modeling coupled with computational fluid dynamics (CFD) methods allows for the prediction of the movement and targeted adhesion of drug-carrying nanoparticles inside the bloodstream. This method discusses treatment strategies instead of the usual methods, with less time and cost to optimize, develop, and design unique drug carriers^7^. Alishiri et al.^8^ have studied targeted drug delivery on the carotid atherosclerotic plaque in two dimensions by nano-liposomes under an external magnetic field and investigated the adhesion of microbubbles to atherosclerotic plaque. Their research showed that the Halbach arrangement of the magnet and changing its location opposite the flow’s direction could enhance drug delivery^9^ used the Euler–Lagrange approach and finite element method (FEM) to simulate the dominant forces, magnet and drag forces, on the particles by defining the link between the Oscillatory Shear Index (OSI) and the efficiency of drug delivery in a particular magnetic field; it was found that areas with a lower OSI are suitable for locating the magnet. The correct magnet layout led to more particles being directed to the target branch by about 4%. Shamloo et al.^10^ studied microbubbles in targeted drug delivery to arrest abdominal aortic aneurysms (AAA). They showed that targeted drug delivery at positive blood flow velocities with microbubbles to the inner part of AAA deteriorated due to the low surface density of MBs. Lindemann et al.^11^ represented a 3D model of 8 artery branch combinations based on FEM to study magnetic gradient and density effects and the distance of the magnet to the geometry. Gul et al.^12^ simulated a two-phase model coupled with the magnetic nanoparticles dynamic model in the bloodstream. They evaluated the influence of the Reynolds number on blood flow topology, drag and magnet force effects, and the position of the magnet on drug delivery. Amani et al.^13^ considered the forces between the particles and the external forces of the magnet, changed the metal to SiO2, Fe3O4, NiO2, silver, and gold, and investigated the effect of particle density on drug delivery. They also studied the relationship between the surface density, a crucial parameter that quantifies the number of particles adhered to the plaque per unit surface area, and the number of particles injected. As the number of large particles injected into the artery with a diameter of 800–1000 nm increased, the surface density increased by about 50% compared to the particles with a 400–600 nm diameter. In general, the effects of geometry and biophysical factors were more influential than particle density in determining the surface density.

Recently, utilizing nano-microcarriers for targeted drug delivery has been considered an alternative treatment for vascular diseases. Researchers have studied different carriers to deliver drugs to the target tissue during this period. Yang et al.^14^ examined the desired inflamed tissue in interaction with the drug in a sample of AAA disease in a type of rabbit by placing the Interleukin-8 drug on the surface of a microbubble by applying an acoustic field and delivering drugs through these particles. In the study, they observed a decrease in the amount of inflammation and a decrease in the amount of drug in the plaque, which indicates the positive effect of this type of treatment on controlling the stability of the plaque.

The synthesis of small-scale metal–organic frameworks (MOFs) has led to a focus on medical and drug delivery (especially in recent years), in addition to their many applications in industrial matters^15^. The major advantage of MOFs is their significant porosity, which makes their weight percentage (wt%) much higher than similar specimens. As a result, the loading rate of the drug is greater than the weight of each nanocarrier (NC), and the drug's transport efficiency per number of carriers increases^16^. Ligands can be coated on MOFs to ensure that adhesion is proportional to the receptor of the targeted tissue cells^14^. Xiao et al.^17^ employed a member of the specific MOF branch known as Zeolitic Imidazolate Frameworks (ZIFs), specifically Zn metal-based ZIF-90, for drug delivery to cancer tissue. Several MOFs, such as ZIF-8, expedite this process due to the acerbic environment near atherosclerosis and cancer tissues. Targeted drug delivery is progressive using MOFs and their response to conditions such as pH and external forces such as magnetic fields.

Moreover, numerous computer simulations are presently employed to design and enhance novel adsorption processes for drug delivery systems. Running computer simulations involves representing a real system and processes using ZIF-8 as a suitable framework. This framework’s surface and other relevant factors are closely associated with the diffusion of large molecules in confined spaces. Both experimental and computational perspectives have already investigated these aspects^17,18^. Zhao et al.^19^ inserted Fe3O4 magnetic particles into MOFs like UIO-66, which formed a composite suitable for drug delivery and cancer diagnosis using magnetic resonance imaging. Earlier studies have demonstrated the use of magnetic fields in targeted drug delivery. Previous research has shown active field applications, such as magnetic fields, in targeted drug delivery^20,21^.

The present study explores drug delivery by MOFs to the inner surface of atherosclerosis within the carotid artery. The performance of MOFs relies on the surface density of nanocarriers through ligand-receptor connections and the parameter toxicity, which is determined by calculating the number of particles that fail to reach the plaque tissue and leave the carotid artery through outlets to other non-target tissues in the body, and the amount of drugs delivered based on the drug capacity of each NC which is simulated by the Bias-Monte Carlo (CBMC) simulations method in Material Studio software. This study uses patient-specific geometries, considering the boundary conditions analogous to the reality of the human carotid (pulsatile blood velocity and pressure) and non-Newtonian blood properties. The external magnetic field effects on the delivery of MOFs to the target wall have been investigated by placing an external magnet with a Halbach arrangement on the outer skin of the patient's neck.

## Materials and methods

### Model geometry

The CT-scan images of a 58-year-old male patient were acquired from Shariati Cardiovascular Hospital, Tehran, Iran, aiming to study targeted drug delivery in the carotid artery to reduce lipid plaque accumulation. The procedure was carried out according to relevant guidelines and regulations approved by Shariati Heart and Vascular Hospital, and informed consent was obtained from the patient.

**Figure 1:**
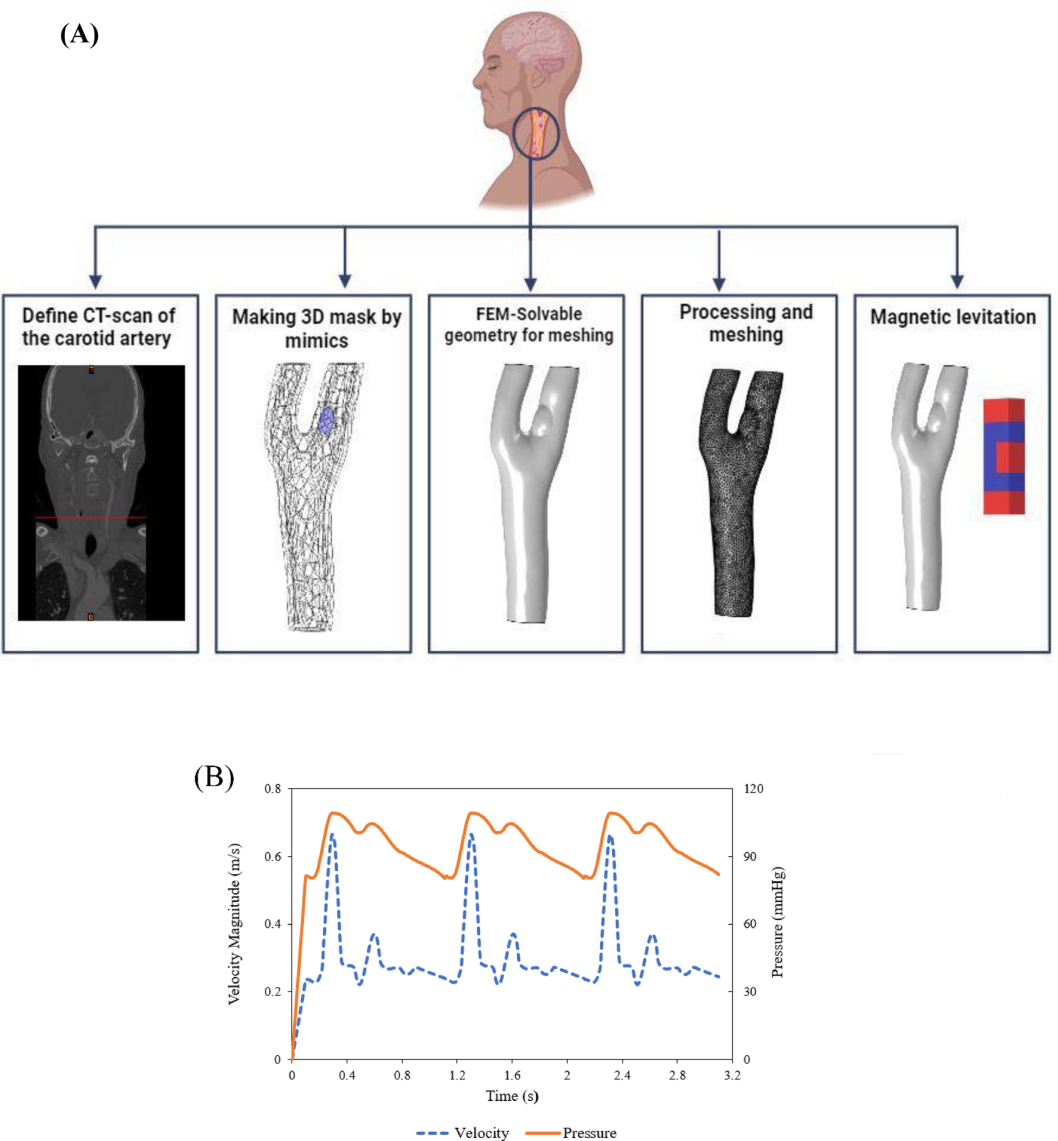
(**A**) The steps of preparing the geometry of the atherosclerotic carotid artery for simulation (i) A CT scan of a patient with atherosclerosis in the Mimics software (ii) Two carotid artery branches were extracted from the two-dimensional CT scan images in three-dimensional form. The extra capillaries were removed, and the external surface of the vessel was smoothed. (iii) The geometry of the carotid artery has been entered into the nonlinear finite element code. (iv) The triangular mesh is applied to the desired geometry. (v) The carotid artery is placed under the Halbach magnetic, and the amount of drug transferred and the surface adhesion of the nanocarriers are compared to the state with and without the magnet. (**B**) The inlet velocity and outlet pressure profiles in three cardiac cycles.

Since geometry is the main factor in the simulation process, including blood flow velocity, transferring particles carrying the drug, and the amount of adhesion to the wall, the carotid artery geometry was constructed by Mimics software using CT scan images accurately. It should be noted that additional capillaries were removed for precision and high-quality meshing and to investigate the effect of the main flow on the artery. According to Fig. 1A, the geometry of the brain carotid artery has a bifurcation with diameters of 1.61 mm and 1.48 mm for each outlet region. The lipid plaque is asymmetric, with a carotid stenosis percentage of 50% in the internal carotid, which is the most exposed part of the plaque.

### Fluid properties and boundary conditions

In the blood flow simulation of the carotid artery, blood was considered incompressible, non-Newtonian, and the fluid regime was laminar. The Carreau–Yasuda model is used to calculate the blood viscosity amount, which is a non-linear relationship between blood shear stress and shear flow rate. According to this model, blood viscosity is obtained from the following equation^22^.

1 $${\eta }_{app}={\eta }_{\infty }+\left({\eta }_{o}-{\eta }_{\infty }\right){\left[1+{\left(\lambda \dot{\gamma }\right)}^{2}\right]}^{\left(a-1\right)/2}.$$

The values of these parameters, according to the previous research, are ${\eta }_{\infty }$ = 0.00345 [Pa s], ${\eta }_{o}$ = 0.161 [Pa s], $\lambda$ = 39.418 [s], and a = 0.479^23^.

The flow regime is laminar and follows mass and momentum equations conservation. Generalized Navier–Stokes equations are used to investigate the effect of dynamic viscosity modeling on non-Newtonian blood flow. Eq. (2a) shows the conservation of mass^24,25^.

2a $$\nabla \cdot u=0.$$

Equation (2b) represents the conservation of momentum.

2b $$\rho \frac{\partial u}{\partial t}+\rho u\cdot \nabla u=-\nabla p+\nabla \tau +F$$

In the above equation, $\rho$ is the fluid density, and $u$ is the fluid velocity, a heart rate function. $p$ is thermodynamic pressure, $F$ is the volumetric force exerted on the fluid, and $\tau$ is the shear tensor dependent on the fluid movement.${S}_{ij}$ is a function of the strain rate_,_ and the Kronecker delta ${\delta }_{ij}$ is defined as follows^26^.

3a $${\tau }_{ij}=2{\eta }_{app}{S}_{ij},$$

3b $${S}_{ij}=\frac{1}{2}\left(\frac{\partial {u}_{i}}{\partial {x}_{j}}+\frac{\partial {u}_{j}}{\partial {x}_{i}}\right)-\frac{1}{3}\frac{\partial {u}_{k}}{\partial {x}_{k}}{\delta }_{ij},$$

3c $${\delta }_{ij}=\left\{\begin{array}{l}1 \quad\,if\, i=j\\ 0 \quad\,if \,i\ne j\end{array}\right..$$

In this simulation, the artery’s inlet velocity and outlet pressures are considered pulsatile based on the heart rate cycle shown in Fig. 1B.

### Forces acting on the drug carriers

During blood circulation in a system containing drug-carrying nanoparticles and blood fluid, two types of volume and surface forces apply to the system. Volumetric forces include the magnetic force (${{\varvec{F}}}_{m}$) caused by a permanent magnet, the gravity force, and the buoyancy force. Generally, during the treatment process, the patient lies horizontally on the bed, so gravity and buoyancy forces are perpendicular to the direction of blood flow. Surface forces include the drag force (${{\varvec{F}}}_{D}$), which is applied to the particle in the opposite direction of the particle velocity, the lift force (${{\varvec{F}}}_{L}$), which affects the particles due to the creation of a pressure gradient in the direction perpendicular to the velocity flow and the particle–particle interaction forces (${{\varvec{F}}}_{p-pi}$). While the diameter of nanoparticles is usually greater than 50nm, the Brownian force is ignored^11,27,28^. The effect of these forces on the magnetic nanoparticles carrying the drug is calculated according to the momentum equation as follows.

4 $$\frac{d}{dx}\left({m}_{p}{\varvec{v}}\right)={{\varvec{F}}}_{D}+{{\varvec{F}}}_{L}+{{\varvec{F}}}_{m}+{{\varvec{F}}}_{p-pi}.$$

In the above equation, ${m}_{p}$ is the particle’s mass, and ${\varvec{v}}$ is the particle’s velocity. Blood fluid from the Eulerian point of view and drug-carrying nanoparticles from the Lagrangian point of view were considered in continuous and dispersed frameworks, respectively, to perform the simulation.

### Particle tracking for fluid flow

The injection times were adjusted from seconds to 1-s intervals according to the cardiac cycle in random and ramp-function manners. Diffusion and specular reflection were used since the particles collided with the surface of the artery wall, and the wall’s roughness significantly affected particle movement and adhesion. In this method, the probability of the particles hitting a tangential surface is 0.5, and that particle’s impact angle at the reflection angle is equal to the initial impact angle.

5a $${q}^{\prime}=q,$$

5b $${v}^{\prime}=v-2\left(n\cdot v\right)n.$$

The particle position vector before contact is ($q$) and after contact is (${q}{\prime}$), the normal surface vector is ($n$) and the particle velocity vector before contact is ($v$) and after contact is (${v}{\prime}$).

Since the particle is reflected in a heterogeneous manner, the velocity vector from the reflection surface can be obtained from Knuden’s Cosine. Furthermore, the position of the particle is calculated according to the law of cosines in three dimensions according to the following equations^29^.

6a $${v}_{t1}=\left|{{\varvec{v}}}_{c}\right|{\text{sin}}\theta {\text{sin}}\varnothing ,$$

6b $${v}_{t2}=\left|{{\varvec{v}}}_{c}\right|{\text{sin}}\theta {\text{cos}}\varnothing ,$$

6c $${v}_{n}=\left|{{\varvec{v}}}_{c}\right|{\text{sin}}\theta .$$

Output tangential velocities are (${v}_{t1}$) and (${v}_{t2}$), the instantaneous velocity of the particle hitting the surface is (${{\varvec{v}}}_{c}$), and the normal velocity is (${{\varvec{v}}}_{N}$). The angle $(\varnothing )$ interval is chosen between $0 - 2\pi$ randomly, and the angle ($\theta$) is obtained from the following equation.

7 $$\theta ={\text{sin}}^{-1}\left(\sqrt{\Gamma }\right)$$

The probability distribution of the normal velocity component, which is called ($\Gamma$), has the above cosine relationship with the angle ($\theta$), which is a random number. The differential equations governing the physics of the problem are based on laminar flow and were solved using the nonlinear unsteady finite element that follows the Algebraic Multigrid. In order to track the particles, the generalized minimal residual solver was used. Furthermore, in this time-solving problem, the Backward Differentiation formula with quadratic accuracy and, finally, a fixed damping factor were chosen as nonlinear methods for damped Newton iterations^8,30^.

**Figure 2:**
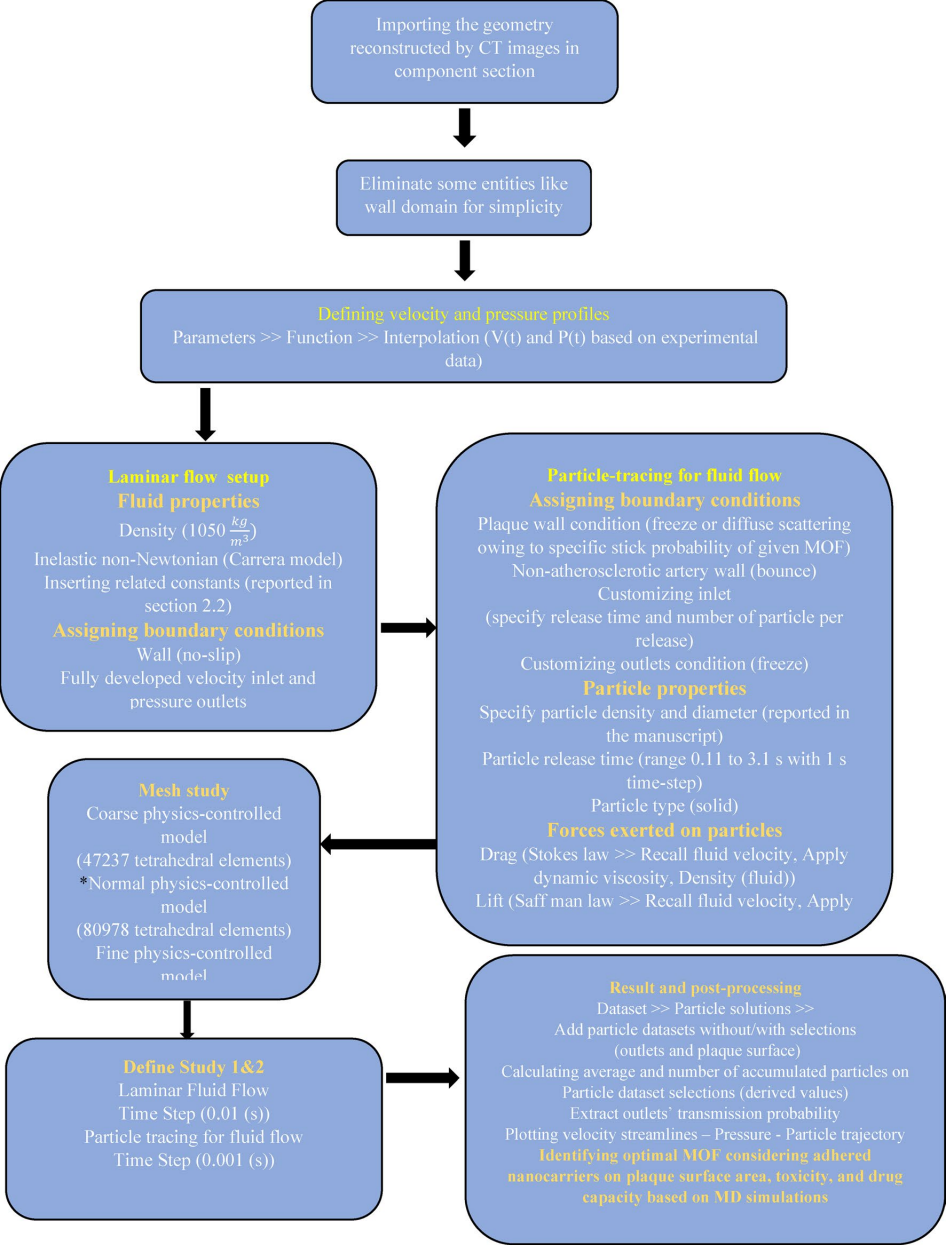
Schematic of steps taken for the patient-specific carotid stenosis simulation providing information on fluid flow and particle tracking setups.

A schematic chart illustrates the simulation procedure, encompassing various steps such as geometry importing, software setup, defining fluid and particle properties, setting particle interactions, establishing boundary conditions, discretization and time step size, and applying forces (Fig. 2).

### Computational method and molecular models

Five MOFs named ZIF-8, ZIF-90, PCN-224, MOF-801 (zr), and UIO-66 used in this study. A cubic cell with dimensions of 61.964 Å, possessing a BET surface area of 1843.4 ± 0.2 m^2^/g, are employed. This cell was derived from the ZIF-8 model, which comprised a ZIF-8 basic center cubic (BCC) cell^31^.

#### Drug loading simulation

In this study, Configurational Bias-Monte Carlo (CBMC) simulations within the sorption module of materials studio software to ascertain the quantity of losartan potassium molecules are employed that were adsorbed into ZIF-8 and four others within MOFs, according to Supplementary Tables 2 and 3 under conditions of 1 bar and 37 °C (310 K)^31–33^. Prior to conducting the CBMC simulations, the torsion degrees of freedom for each individual losartan potassium molecule^34^. The interactions between losartan potassium and ZIF-8 were characterized by employing Lennard–Jones (L–J) 12–6 and Coulomb potentials^34^. The L–J parameters for both the ZIF-8 and losartan potassium molecules were determined using the Universal Force Field (UFF)^31,34–36^.

An atom-based summation approach with cubic spline truncation and the cutoff distance of 13.5 Å to account for Van der Waals interactions are employed. To estimate the atomic charges of MOFs, the Extended Charge Equilibration (EQeq) method is utilized. Additionally, electrostatic interactions were measured using the Ewald summation method, which ensured an accuracy of 10^−4^ kcal/mol^31,34^. Both the equilibration and production steps were conducted for 106 cycles each. For a more comprehensive understanding of the CBMC simulations, please consult Supplementary Table 3.

After conducting CBMC simulations, we successfully determined the absorption capacity of ZIF-8 and other MOFs for a specific drug under controlled pressure and temperature conditions. The $wt\%$ formula is calculated as follows^34^.

8 $$wt\%=\frac{weight \, of \, loaded \, drug \, in \, each \, nanocarrier }{weight \, of \, each \, nanocarier+weight \, of \, loaded \, drug \, in \, each \, nanocarrier}\times 100.$$

This crucial information serves as a foundation for subsequent investigations into drug delivery mechanisms. In order to comprehensively evaluate the release behavior of the drug within MOFs, molecular dynamics (MD), simulations have been employed, a widely utilized technique in the study of drug delivery system^37^. The initial configuration for these MD simulations was established based on the MOF’s minimum energy state^38^. Given the inherent mobility of guest molecules within MOFs and the dynamic nature of drug release processes, the accurate representation of real systems through simulation becomes imperative.

Furthermore, previous studies have indicated the necessity of conducting flexible MOF simulations when the size of the guest molecule closely matches that of the MOF pore. In light of this, we employed the Forcite module of Materials Studio to carry out MD simulations for the MOFs. The Universal Force Field (UFF) was utilized in these simulations to account for bond stretching, angle bending, and dihedral torsions, as implemented within Materials Studio^31^. The displacement of rigid MOF structures through a series of geometry optimization steps is systematically optimized. The optimization process was iterated until achieving energy convergence at a threshold of 0.0001 kcal/mol, as evidenced by the results presented in Supplementary Table 3. It is worth noting that the cell geometry remained unaltered during the optimization step. Following this optimization procedure, MD simulations were conducted within the NVT ensemble, employing a time step of 1 fs.

#### Carrier adhesive dynamics model

According to the previous studies^34,39^, P-selectin aptamers have a high adhesion ability to plaque tissue. They can establish a better bond with the target receptors compared to other antibodies. Deccuzi and Ferrari’s^40^ model calculates the adhesion probability function. Based on this model, the force and torque that the fluid exerts on the desired particles are calculated using the following equations:

9 $$F=3\pi {d}_{p}l{\eta }_{app}S{F}^{S},$$

10 $$T=\frac{1}{2}\pi {d}_{p}^{3}{\eta }_{app}S{T}^{S}.$$

${F}^{S}$ and ${T}^{S}$ are fixed values depending on the aspect ratio $\left(\gamma =\frac{a}{b}\right)$. In the mentioned equations, $l$ is the particle’s distance from the substrate. Furthermore, particle size (${d}_{p}$) and wall shear stress (${\eta }_{app})$ and $S$ are among the determining parameters. The aspect ratio value in spherical particles is equal to $1$, and the constant values ${F}^{S}$ and ${T}^{S}$ in the equations, according to the results of Goldman et al.^41^, are reported as $1.668$ and $0.944$, respectively. The probability of forming at least one ligand-receptor bond, which is the same adhesion probability, is called (${P}_{\alpha }$) for particles and is calculated from the following formula (Eq. 11). According to the definition, the higher the value of (${P}_{\alpha }$), the more adhesion the particle will have to the substrate.

11 $${P}_{a}=\pi {r}_{0}^{2}{m}_{r}{m}_{l}{K}_{a}^{0}\times {\text{exp}}\left[-\frac{\beta {d}_{p}{\eta }_{app}S}{{K}_{B}T{r}_{0}^{2}{m}_{r}}\left[3\left(\frac{{d}_{p}}{2}+{\delta }_{eq}\right){F}^{S}+\frac{{d}_{p}^{2}}{{r}_{0}}{T}^{S}\right]\right].$$

The circular cross-section radius of the particle ($${r}_{0}$$) is the distance ($${h}_{0})$$ that it is located from the bed, and $${h}_{0}$$ is also the maximum distance between the particle and the vascular wall so that the receptor and ligand bond can be formed. $${m}_{r}$$ and $${m}_{l}$$ show the surface densities of receptors and ligands, respectively. $${K}_{a}^{0}$$, binding constant, is defined in a state where the load of the ligand-receptor bond is equal to zero. $$\beta $$ and $${K}_{B}T$$ are reactive compliance and Boltzmann thermal energy, respectively. The equilibrium distance ($${\delta }_{eq}$$) is the distance between the spherical particle and the vascular bed.

The most important factor for particle adhesion is average shear stress (${\eta }_{app}$ S)^39^, whose value can be calculated on the surface of the plate according to the pulsation of the flow and fields such as the magnetic field that lead to the creation of volume forces. After placing (${\eta }_{app}$ S) in Eqs. (9) and (10) to calculate the probability function (${p}_{\alpha }$), we will only need the particle diameter value because the remaining constant coefficients have been obtained from previous molecular dynamics studies for the aptamer P-selectin (Supplementary Table 4).

Other particle parameters (such as density) will be used to calculate the surface density and the number of particles that hit or stick to the plate surface. The results will be reported for different carrier diameters and types. The results of previous research, such as^40^, indicate that adhesion can be increased in addition to improving the affinity coefficient. However, the adhesion amount directly relates to ligands and receptors’ surface densities. The NCs used in this study carry losartan potassium; in other words, this drug is loaded inside them. In order to apply magnetic properties, a magnetic core must be placed inside the carriers during the synthesis, which is usually made of Fe3O4. The embedding process can be done for MOFs^8,19^ and causes the particles to deviate toward the magnet when a magnetic field is established. In addition, ligands are coated on these NCs so that the drug can be delivered to the atherosclerosis tissue in a targeted manner by establishing affinity. The more accurate targeted drug delivery is, the more it prevents unnecessary drug circulation in the bloodstream, which makes it possible to reduce the waste of the drug or its absorption by healthy organs (and possibly the resulting complications)^42^.

## Results and discussions

### The number of injected nanocarriers based on adhesion and toxicity

**Figure 3:**
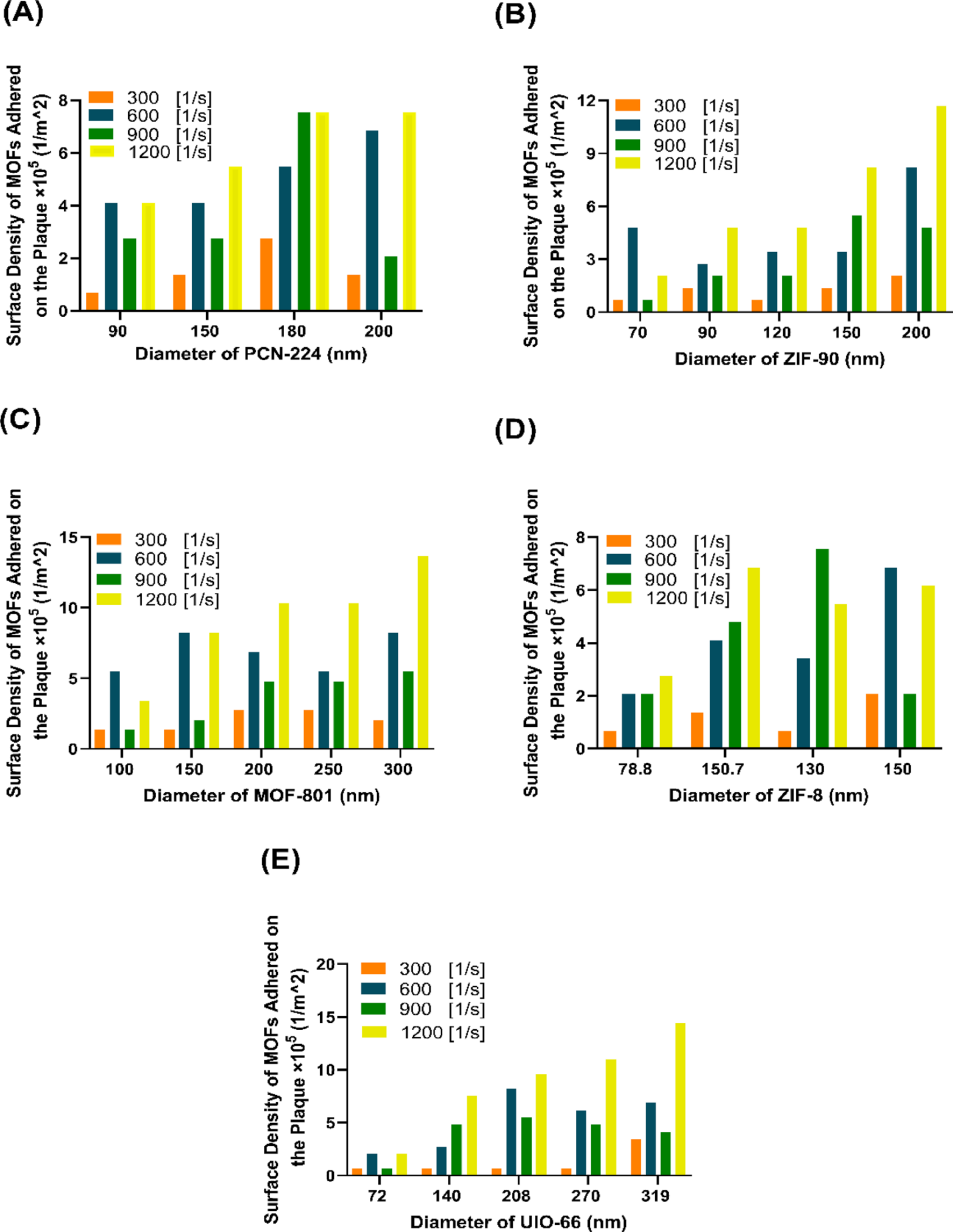
The surface density attached to nanocarriers with different injection numbers and sizes. (**A**) PCN-224 nanocarriers for diameters of 90 nm, 150 nm, 180 nm, and 300 nm; (**B**) ZIF-90 nanocarriers for diameters of 70, 90, 120, 150, and 200 nm; and (**C**) MOF-801 nanocarriers for diameters of 100 nm, 150 nm, 200 nm, 250 nm, and 300 nm. (**D**) ZIF-8 nanocarriers for diameters of 78.8 nm, 105.7 nm, 130 nm, and 150 nm. (**E**) UIO-66 nanocarriers for diameters of 72 nm, 140 nm, 208 nm, 270 nm, and 319 nm. In all nanocarriers, at larger diameters and with more inlets, the amount of surface density of nanocarriers adhered to the plaque is the highest.

The NC’s influential adhesion parameters to the inner wall of atherosclerosis inside the artery, the velocity of blood flow entering the carotid artery, and the direction and magnitude of the magnetic field (Supplementary Note 6), depend on the number of NCs entering each cardiac cycle and the mechanical properties of the NCs (diameter and weight density). The NCs surface density value for different numbers of particle injections in each cardiac cycle and different diameters is shown in Fig. 3. In most instances, a higher influx of nanocarriers into the artery is observed to correspond to an increased surface density count. However, it can be said that in most cases, the inlet number of 600 NCs per cardiac cycle has an acceptable level of surface density (less than 40%) (less than 40%) compared to the higher inlet number of NCs (e.g., 1200). The distribution of NCs in the carotid artery area shows that the presence of NCs in the bifurcation areas is more due to low and return fluid flow compared to the areas close to the carotid branch exits**.** Due to the non-linearity of modeling, including asymmetric geometry, non-Newtonian blood, different types and diameters of NCs, and pulsatile velocity and pressure, NCs tracking inside the carotid artery and the number of surface density assessment based on the number of different NC injections is complicated. However, in a previous study^13^, in which they investigated the amount of surface density in the coronary artery with different diameters and a different number of solid particles, it was observed that increasing the number of inlet particles, in most cases, increases the amount of surface density of particles adhered to atherosclerosis, which is compatible with the present study.

**Figure 4:**
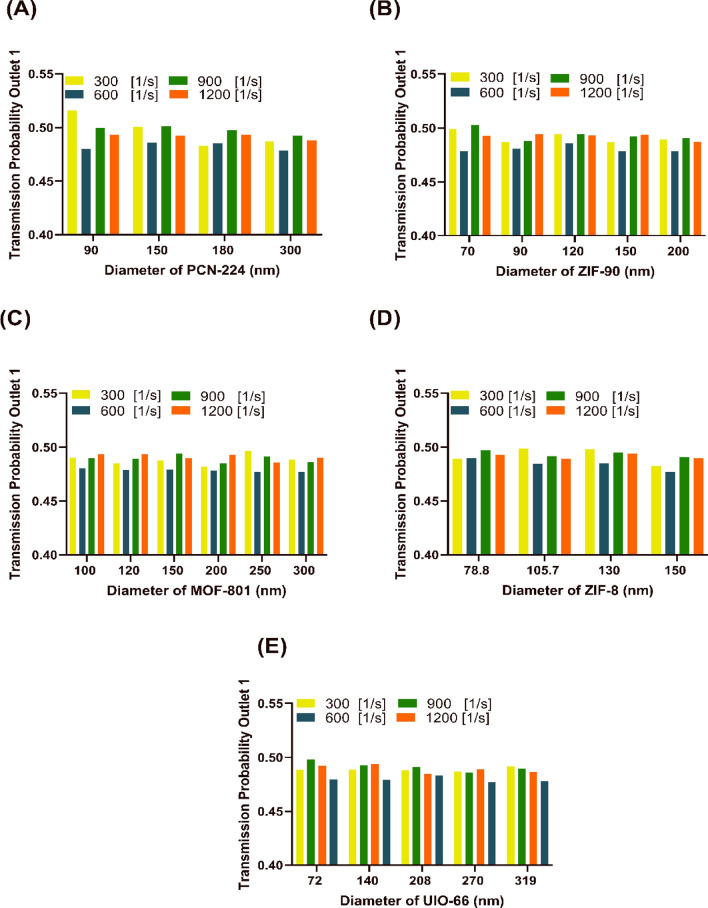
Transmission probability of nanocarriers to the internal carotid for different sizes of nanocarriers (**A**) PCN-224 nanocarrier, (**B**) ZIF-90 nanocarrier, (**C**) MOF-801 nanocarrier, (**D**) ZIF-8 nanocarrier, (**E**) UIO-66 nanocarrier. It is seen that with 600 nanocarriers entering all diameters of nanocarriers, the probability of producing toxicity in the internal carotid artery is the lowest.

Another prominent factor in determining the optimal delivery of NCs to the target wall is checking the extent of their penetration into the downstream areas of the artery. Figure 4 analyzes the number of particles passing through outlet 1. The important point is that the more particles that pass through the outlet, the greater the possibility of toxicity to the brain caused by drugs loaded on the brain. According to Supplementary Fig. S4, after performing the t-test, it can be seen that the average number of 600 particles (blue dots) passing through outlet 1 is much less than the others for different diameters, and it is around 0.48 transmission probability. On the other hand, calculating the p-value, which is less than 0.05, shows a significant difference between the acquired transmission probability data for a 600-particle injection compared to 1200 particles. Statistical analysis shows that in most MOFs, considering the standard deviation (SD) factor, the number of 600 particles compared to 1200 particles for different diameters takes less outlier data, which can be a factor in considering the optimal value. Therefore, the optimal release of the 600 NCs (per cardiac cycle) at the inlet causes the lowest possible toxicity and injury to other parts of the patient’s body and has sufficient efficiency compared to the large number of entering NCs, resulting in an increasing the surface density.

Generally, the greater the number of NCs entering the artery, the more likely they are to pass through the carotid outlet. Also, a reduction in the number of NCs causes a drop in the possibility of NCs hitting the inner wall of atherosclerosis. One of the main reasons is the blood flow velocity profile and the equilibrium position of the particles inside the channel, which are caused by the lift force and the near-wall shear stress^43,44^.

As indicated by the results depicted in Fig. 3, it is observed that the surface density for each MOF is either the highest or, at the very least, the second-highest when considering the maximum diameter within that specific carrier type, exemplified by the 200 nm ZIF-90. This trend can be attributed to two key factors. Firstly, in accordance with Eq. (11), the adhesion probability of particles demonstrates a direct and ascending relationship with the particle diameter. This implies that an increased diameter of a specified carrier enhances the likelihood of particles adhering to the plaque tissue when there is a collision between them, thereby resulting in elevated surface density.

Secondly, in addition to the adhesion probability, the analysis must account for other contributory factors, prominently the drag force. As detailed in Supplementary Note 1.3, the drag force experiences augmentation with an increase in nanoparticle diameters. This augmentation holds considerable implications for the trajectory of nanocarriers, notably influencing the carrier velocity. The heightened drag force extends the carrier’s transit time, allowing for increased interactions with other forces, such as lift forces and particle–particle interactions, facilitating the carrier's arrival at the plaque tissue.

Acknowledging the inherent nonlinearity of our study, a strictly linear relationship between surface density and diameter for each MOF is not universally applicable. Particularly where diameters closely approximate each other, predicting the relationship between surface density and diameter is challenging, with observed patterns exhibiting both ascent and descent. This nonlinear behavior is likely influenced by factors such as the impact of additional forces (e.g., lift force, particle–particle interaction), as well as the non-asymmetric geometry. While the lift force may have a diminished impact on particle tracing, its significance arises in scenarios where other forces balance each other, and its direction varies with the particle’s relative velocity to the fluid. That is, according to Saff man (Supplementary Note 1.2), in situations where the fluid velocity is higher than that of the particle, which commonly occurs in areas farther than the central parts of the flow, we have a lift force directed toward the wall, and vice versa. Additionally, the geometry plays a pivotal role in this nonlinearity: particle trajectories initiate from the inlet, progress alongside the artery, encounter bifurcations, and subsequently enter outlets, with pathlines influenced by interactions with the fluid and collisions with the artery wall before reaching the outlet branch.

### Drug delivery performance of nanocarriers

**Figure 5:**
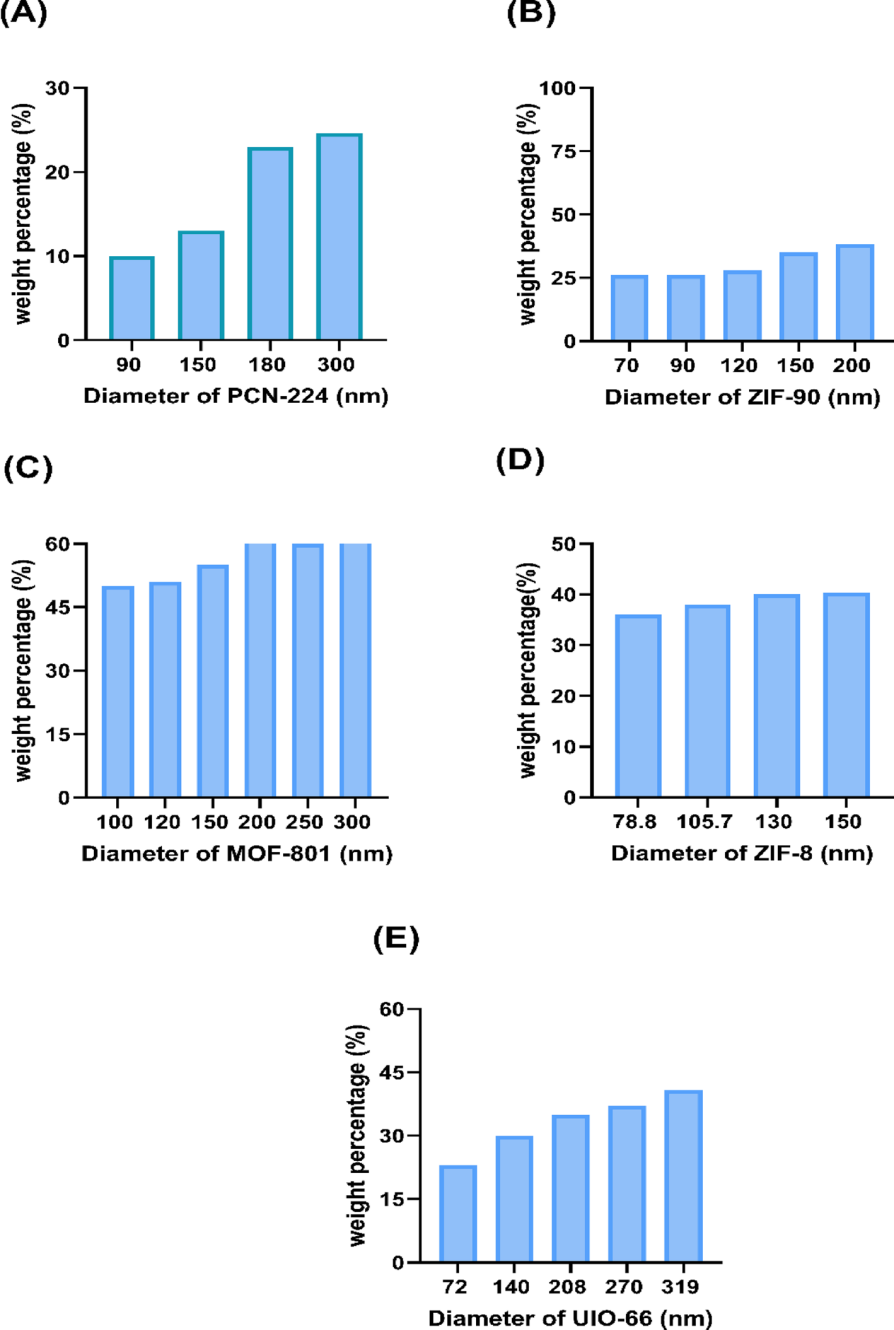
The wt% of nanocarriers with different sizes of each nanocarrier. (**A**) PCN-224 nanocarriers for diameters of 90 nm, 150 nm, 180 nm, and 300 nm; (**B**) ZIF-90 nanocarriers for diameters of 70, 90, 120, 150, and 200 nm; and (**C**) MOF-801 nanocarriers for diameters of 100 nm, 150 nm, 200 nm, 250 nm, and 300 nm. (**D**) ZIF-8 nanocarrier for diameters of 78.8 nm, 105.7 nm, 130 nm, and 150 nm. (**E**) UIO-66 nanocarriers for diameters of 72 nm, 140 nm, 208 nm, 270 nm, and 319 nm. In all nanocarriers, at larger diameters and with more inlets, the amount of surface density of nanocarriers adhered to the plaque is the highest.

Another key factor in the design of the type and size of drug NCs that should be considered is the amount of drug delivered to the target wall. MOFs play a very prominent role in the amount of drug loading capacity and the amount of drug delivery to the target wall. The loading NCs capacity and physical properties are represented in Fig. 5 and Supplementary Table 5 as well. Here, the amount of drug delivery to the target wall is obtained using the following equation:

12 $$\text{Transferred drug }=\text{ surface density}\times \text{weight of loaded drug}.$$

**Figure 6:**
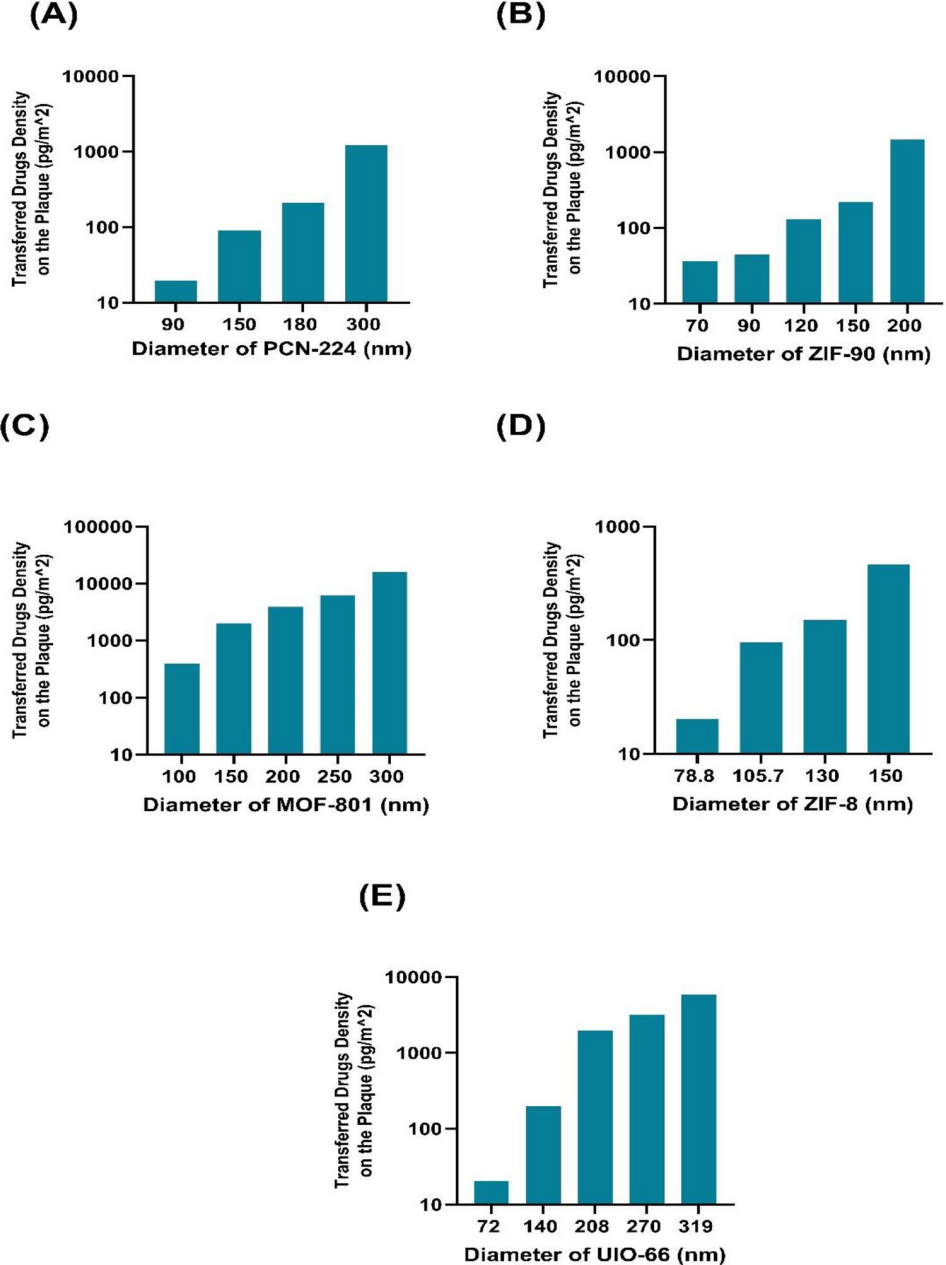
The density of the transferred drugs to the plaque for different sizes of nanocarriers with the optimal inlet number of 600 nanocarriers (**A**) PCN-224 nanocarrier, (**B**) ZIF-90 nanocarrier, (**C**) MOF-801 nanocarrier, (**D**) ZIF-8 nanocarrier, (**E**) UIO-66 nanocarrier.

To determine the transfer of drugs to the lipid plaque region, we can use Eqs. (6a) to (6c). This equation takes into account the mass of MOFs and wt%, which are both factors that influence drug transfer. The amount of drug transferred to the inner wall of atherosclerosis in different types and sizes of NCs for the inlet number of 600 NCs injection per cardiac cycle is shown in Fig. 6. Moreover, by increasing the NC’s diameter, the rate of drug transfer to the plaque wall increases. Among the MOFs, MOF-801 has the highest drug delivery rate to the target, which can be introduced as an optimal drug carrier for delivering the drug to the inner wall of the atherosclerotic plaque inside the carotid artery. This phenomenon occurs because, according to Eq. (12), the transferred drug also increases with an increase in the weight of the loaded drug.

### Effect of magnetic field on drug delivery

One of the ways to increase the performance of drug delivery and target the direction of NC’s movement is by using a magnetic field with high efficiency. In this study, we have used the Halbach arrangement in the optimized placement position from the previous study^8^, in which magnetic nanoparticles (${\text{Fe}}_{3}{\text{O}}_{4}$) with a diameter of $50\text{ nm}$ are placed inside each of the NCs, to investigate the delivery of the drug carrier to the carotid artery under the magnetic field.

**Figure 7:**
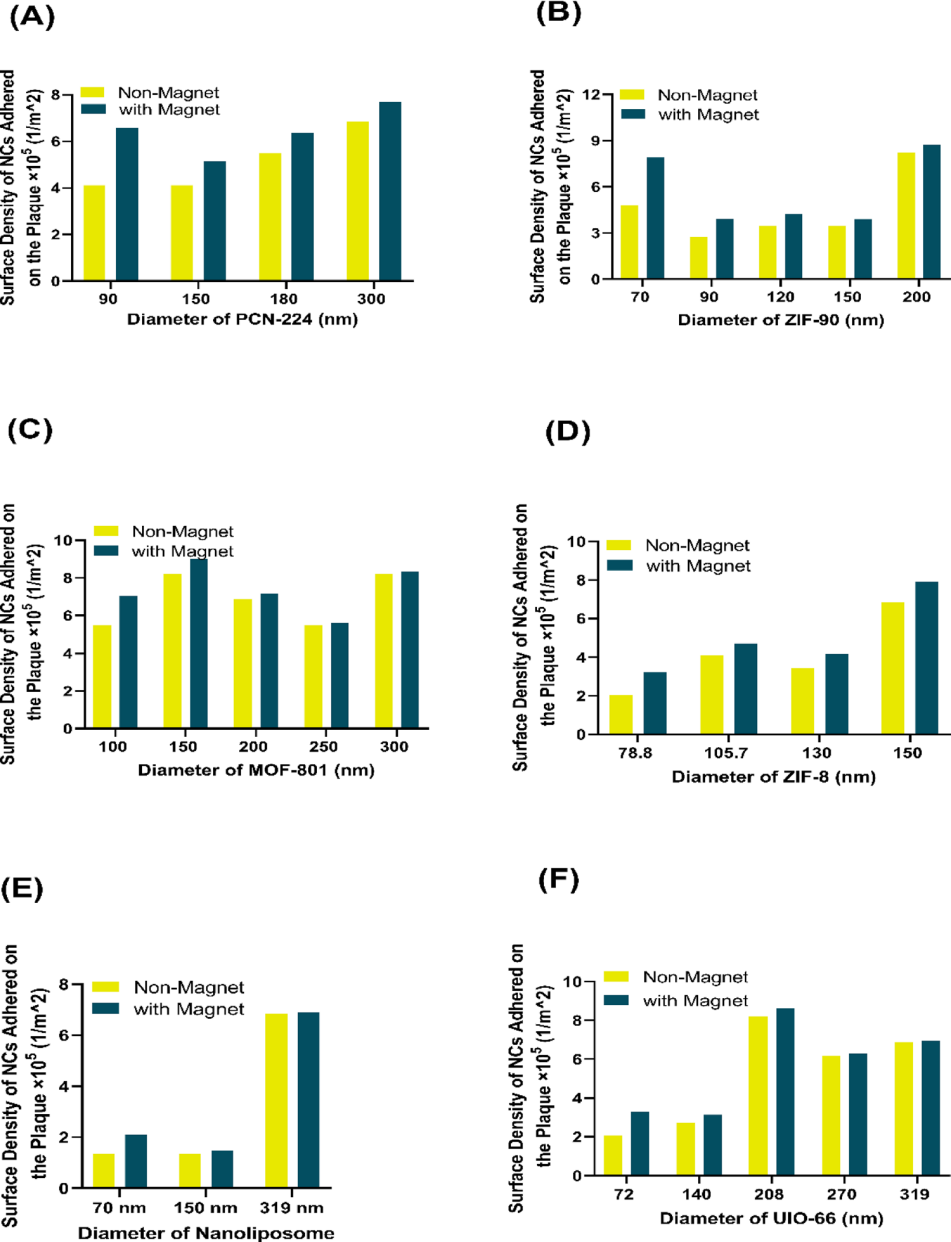
Comparing the surface density of nanocarriers adhered to the plaque with the optimal inlet number of 600 nanocarriers with and without a magnetic field. (**A**) PCN-224 nanocarrier, (**B**) ZIF-90 nanocarrier, (**C**) MOF-801 nanocarrier, (**D**) ZIF-8 nanocarrier, (**E**) UIO-66 nanocarrier. The presence of a magnetic field increases the number and surface density of nanocarriers adhered to the plaque compared to the absence of the magnetic field.

The amount of surface density inside the carotid artery in both cases with and without applying a magnetic field is shown in Fig. 7. It can be seen that the magnetic field has a better performance for all types of NCs in terms of their adhesion to the plaque wall. However, we observed that by increasing the NC’s diameter, the effect of the magnetic field on the amount of surface density decreased. A significant difference in the amount of surface density is evident in the lower diameter of the NCs. For instance, in PCN-224 with a diameter of 90 nm, by applying a magnetic field, an increase in the amount of surface density can be seen by about 1.5 times, which can be due to the low density of these NCs compared to others. Because the mass of NCs has an opposite proportional relationship to the acceleration of their movement according to Newton’s second law, that can increase the alteration in the direction of movement of NCs inside the carotid artery towards the target branch.

**Figure 8:**
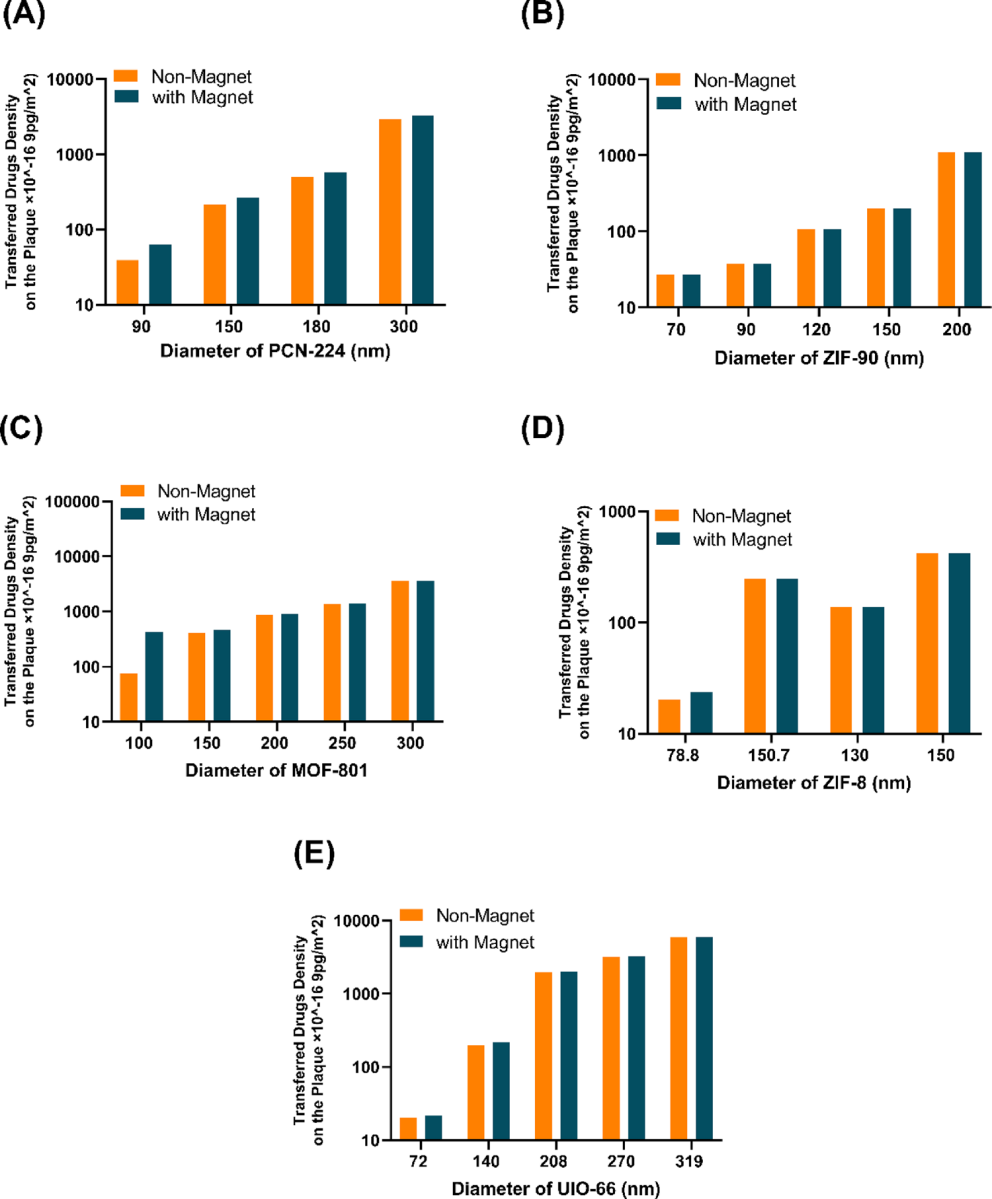
The density of the transferred drug to the plaque with the optimal inlet number of 600 nanocarriers with and without a magnetic field (**A**) PCN-224 nanocarrier, (**B**) ZIF-90 nanocarrier, (**C**) MOF-801 nanocarrier, (**D**) ZIF-8 nanocarrier, (**E**) UIO-66 nanocarrier.

The density of the drug transferred to the inner wall of the atherosclerotic plaque in different types and sizes of NCs, in the absence and the presence of a magnetic field, is shown in Fig. 8. It can be seen that, in most of the NCs, there is not much difference in the density of drugs delivered to the target wall in the presence of a magnetic field compared to the absence of a magnetic field. However, for PCN-224 in a smaller diameter ($90\text{ nm}$), there is a $36\%$ increase, and in a $200\text{ nm}$ diameter, there is a significant decrease of one-third in the amount of transferred drug. The increase in drug delivery in a smaller diameter can be related to their high deviation towards the target and high adhesion to the plaque wall. In the large diameter ($200\text{ nm}$), due to the NCs’ great mass, the magnetic field does not affect their adhesion. Because the magnetic core of $50\text{ nm}$ ${\text{Fe}}_{3}{\text{O}}_{4}$ is embedded in MOFs, the drug loading percentage decreases for specified carriers with constant outer diameters; however, this reduction is negligible in particles with high diameters. Notwithstanding the diminution in capacity associated with the incorporation of a magnet in each MOF, the magnetic force serves to attract nanocarriers toward plaque tissue, thereby augmenting the surface density of nanoparticles. To expound, the parameter governing drug transfer to the tissue is computed through the multiplication of the drug-carrying capacity of each MOF and the surface density of nanoparticles (Eq. 11). The interplay between these two factors, namely the reduction in the drug capacity of MOFs and the escalation in surface density, offsets each other, elucidating the observed similarity in transferred drug quantities among most MOFs.

In this study, we investigated the efficacy of targeted drug delivery to plaque in patients with atherosclerosis using a simulation approach. Our research aimed to compare the performance of five distinct MOFs as drug carrier particles. By employing the simulation, we could analyze and compare the impacts of drug loading, particle dimensions, and the number of particles introduced during each cardiac cycle. This comprehensive analysis provided valuable insights, leading to a more accurate understanding of the treatment process.

## Limitations and future works

The simulation exclusively focuses on the vessel’s geometry with a 40% asymmetric blockage in the inner vessel wall. However, it is essential to note that different geometries of the vessel can impact the flow dynamics and particle collision outcomes. Moreover, the study assumes a laminar blood flow, but in reality, the flow characteristics may vary depending on the vessel’s shape, geometry, and inlet pulsatile velocity, possibly leading to turbulent flow characteristics. Additionally, there is a possibility for errors and uncertainties in particle capturing data in the ligand-receptor bindings processes. It should be acknowledged that particles sometimes detach from the plaque after hitting the wall and rebound, during which the interaction is determined twice, or in a similar situation, one particle can be captured twice while the first collision satisfies the drug delivery of MOFs. Although this simulation yields an exhaustive and accurate result, it falls short of replicating real-world scenarios with absolute precision.

To further enhance the understanding of the subject, future studies can explore different geometries with varying degrees of clogging across different sections of the vessel wall. Additionally, investigating the impact of turbulence effects, turbulence transition, and small vortex structures on surface density adhesion would provide valuable insights. Conducting more simulations would be beneficial to reduce potential errors in particle interactions with the plaque and data collection.

In the current simulation, five different MOFs were examined, aiming to calculate the amount of drug loaded on each. In order to advance this research and find the optimized drug loading amount, it is recommended to modify the drug loading on each MOF by altering the solvent properties, including variations in PBS concentrations, acidity, or alkalinity. Furthermore, considering that different synthesis methods yield MOFs with diverse shapes, such as oval or cubic, it would be worthwhile to investigate their drug delivery capabilities in various shapes.

## Conclusion

In this study, first, by using MD methods and Van der Waals forces, losartan potassium loading was done as an effective drug in different MOFs, and the loading percentage was reported for each nanocarrier. Then, we investigated the targeted drug delivery using five different types of MOFs by simulation modeling of a patient-specific geometry of an artery with atherosclerosis, using FEM and matching the biological conditions of the body with the non-Newtonian properties of blood. The pulsating inlet velocity was applied for better performance and maximum adhesion. The statistical results showed that increasing the MOF’s diameters increases the surface density caused by collision; on the other hand, the highest value of surface density is obtained for 1200 and 600 particle injections per cardiac cycle. Furthermore, taking into account the conditions close to the experimental drug delivery model, it can be seen that some of the MOF drug carriers pass through outlet 1, which leads to the brain. Obviously, to maintain the patient's health and to consider the consequences of the drug damaging the brain, the minimum amount of passing MOF should be considered the optimal case, and the number of 600 particles injected in one cycle has the lowest average probability, approximately 0.48.

Additionally, by comparing different MOFs, the results showed that for 600 particle injection, MOF-801 and UIO-66 transfer the highest amount of medicine to the plaque, respectively. Due to the different inherent properties of these carriers (including density, synthesizable diameter, and wt%), values such as the amount of transferred drug and surface density are unique to them. In addition, these investigations using an external magnetic field to navigate and increase the surface density showed that in comparing and exploring the performance of drug delivery with the help of magnets with the Halbach arrangement, the amount of drug transferred and the surface density increased due to the application of the magnetic force. All these studies have been done to find a more effective, safe, and less invasive disease treatment method. Using MOFs as nanocarriers reduces treatment costs for the patient due to easy synthesis and high drug-loading capacity. It can also facilitate the treatment process due to the lowest level of toxicity and risk for internal tissues, and it can be a suitable tool for treatment in modern medical fields. Our results can benefit the selection of optimal MOFs to design drug carriers to address targeted drug delivery to various carotid artery stenoses.

### Supplementary Information

Supplementary Information.

## Data Availability

The data used in the current study is available from the corresponding author on reasonable request.
