# Author Correction: In‑silico study of drug delivery to atherosclerosis in the human carotid artery using metal–organic frameworks based on adhesion of nanocarriers

**DOI:** 10.1038/s41598-024-58331-0

**Published:** 2024-04-03

**Authors:** Amir Shamloo, Tahoora Naseri, Ali Rahbary, Mohammad Ali Bakhtiari, Sina Ebrahimi, Iman Mirafzal

**Affiliations:** 1 School of Mechanical Engineering, Sharif University of Technology, Azadi Ave., Tehran, Iran; 2 Stem Cell and Regenerative Medicine Institute, Sharif University of Technology, Tehran, Iran

Correction to: *Scientific Reports* 10.1038/s41598-023-48803-0, published online 06 December 2023

The original version of the Article contained an error in the Author contribution statement.

“These authors contributed equally: Amir Shamloo and Tahoora Naseri.”

now reads

“These authors contributed equally: Amir Shamloo, Tahoora Naseri, and Ali Rahbary.”

The original Article has been corrected.

