# Supplementary material for: In-silico study of drug delivery to atherosclerosis in the human carotid artery using metal–organic frameworks based on adhesion of nanocarriers
Source: Sci Rep. 2023 Dec 6;13:21481. doi: 10.1038/s41598-023-48803-0 (PMC10700345; doi:10.1038/s41598-023-48803-0)
Supplement: Supplementary file 1 — Supplementary Information. [file 41598_2023_48803_MOESM1_ESM.docx]

**Supporting Information for**

**In-Silico Study of Drug Delivery to Atherosclerosis in the Human Carotid Artery Using Metal-Organic Frameworks Based on Adhesion of nanocarriers**

Amir Shamloo ^a,b,1,*^, Tahoora Naseri ^a,b,1^, Ali Rahbary ^a,b^, Mohamad Ali Bakhtiari ^a,b^, Sina Ebrahimi^a,b^, Iman Mirafzal^a,b^

**^a^ School of Mechanical Engineering, Sharif University of Technology, Tehran, IRAN**

**^b^ Stem Cell and Regenerative Medicine Institute, Sharif University of Technology, Tehran, IRAN**

**^1^Equal contribution**

*^*^Corresponding Author: Dr. A. Shamloo,* School of Mechanical Engineering, Sharif University of Technology, Azadi Ave., Tehran, IRAN, Tel: 98-21-66165691, Fax: 98-21-66165599, email: [*shamloo@sharif.edu*](mailto:shamloo@sharif.edu)

**Supplementary Notes**

**Supplementary Note 1.1- Magnetic Field**

Magnetic delivery systems create a strong magnetic field outside the body. This power is provided by a permanent magnet and is calculated using Maxwell's equations and the rules of Ampere and Gaussian laws. By creating bipolar torque, paramagnetic nanoparticles align with the magnetic field in response to the continuous magnetic field. Finally, the nanoparticles carrying the drug are absorbed into the plaque (where the magnet is located). The magnetic field's potential is calculated according to Gaussian law [1].

| $\nabla.B=0$ | (S1) |
| --- | --- |

In the above Equation $B$ is the flux density of the magnetic field. The intensity of the magnetic field is obtained from the following Equation.

| $\nabla\times H=J$ | (S2) |
| --- | --- |

According to Ampere's law, $H$ is the intensity of the magnetic field and $J$ is the electric field that has been ignored [2], [3].

| $M=\chi H$ | (S3) |
| --- | --- |

The value of the magnetization vector per volume unit is computed in the **Eq. S3**. In this study, the magnetic intensity, magnetization vector, and vacuum permeability in the clogging branch were related as **Eq. S4** indicates. Based on the experimental results, the magnetic field flux density can be calculated according to **Eq. S5**.

| $B=\mu_{0}(H+M)$ | (S4) |
| --- | --- |
| $B=\mu_{0}\left( 1+\chi\right)H$ | (S5) |

Since the magnetic flux density affects the blood fluid and the walls of the carotid artery, the amount of vacuum magnetic permeability is $\mu_{0}=4\pi\times{10}^{-7}(\frac{N}{A^{2}})$. The magnetic sensitivity of the materials and the amount of magnetic vacuum permeability are indicated in expressions$\chi$ and $\mu_{0}$, respectively. The sensitivity of the magnetic field of materials is calculated according to the following Equation.

| $\chi=\mu_{r}-1$ | (S6) |
| --- | --- |

The density of the magnetic field flux is defined as follows.

| $B=\mu_{0}\mu_{r}H$ | (S7) |
| --- | --- |

The parameter $\mu_{r}$ for the artery texture, diluted blood, and the air is 0.999991, 0.99999095, and 1, respectively. Vector $B$ is used to direct the nanoparticles to the lipid plaque. The existence of clots in the path and under the magnet's location in the narrowing area increases number of particles colliding with the plaque. The force applied to the nanoparticles comes from the subordinate relation.

| $F_{m}=\mu_{r}(m_{np,eff}.\nabla)H_{a}$ | (S8) |
| --- | --- |

In the above Equation $m_{np,eff}$ is the effective dipole moment on nanoparticles. This value is calculated according to **Eq. S9**. When nanoparticles are exposed to a magnetic field, due to the size of the nanoparticles, the magnetic domains are aligned with the direction of the field, and the more the intensity of this field increases, the more domains are in the same direction. Magnetic saturation is the state in which all domains are aligned with the field. for nanoparticles with a volume of is obtained as the following Equation.

| $m_{np,eff}=V_{np}f(H_{a})H_{a}$ | (S9) |
| --- | --- |

The magnetic field vector of nanoparticles ($M_{np}$) is equal to:

| $M_{np}=\chi_{np}H_{in}$ | (S10) |
| --- | --- |

In this Equation ($\chi_{np}$) is the magnetic susceptibility of the particles. In the saturation state of magnetism, the following relationships are assumed. According to **Eq. S11** and **Eq. S12**, the amount of saturation magnetization of nanoparticles with ($M_{snp}$) and the self-demagnetization of nanoparticles with ($H_{demag}$) are displayed. Self-demagnetization is the opposite of ($H_{a}$) and equal to ($\frac{M_{np}}{3}$) [4].

| $M_{np}=M_{snp}$ | (S11) |
| --- | --- |
| $H_{in}=H_{a}-H_{demag}$ | (S12) |

In general, the function f ($H_{a}$) applied to the nanoparticles immersed in the fluid is calculated from the Equation. The maximum magnetic field of the permanent magnet ($H_{a}$) is the simulation of the effective field in the blood fluid in the artery was equal to (191577.65 A/m). The saturation magnetism of these nanoparticles is about 191577.65 (A/m). ${Fe}_{3}O_{4}$ was used as a magnetic nanoparticle embedded in MOFs with a density of $5310 (\frac{kg}{m^{3}})$ and a diameter of $50 nm$.

**Supplementary Note 1.2- Saffman Lift Force**

When a particle is immersed in viscous fluid flow, there is a velocity discrepancy and, subsequently, a discrepancy in pressure between the upper and lower sides of the particle. This difference generates a lift force for particles in a gradient of fluid velocity profile. According to Saffman, the following Equation represents:

$F_{L}=-20.3{d_{p}}^{2}L_{v}\sqrt{\eta_{app}\rho\frac{u-v}{\left| L_{v} \right|}}$ (S13)

$L_{v}=\left| u-v \right|\times[\nabla\times\left( u-v \right)]$ (S14)

The expression $\nabla\times\left( u-v \right)$ in the equation represents the rotational flow field [5].

**Supplementary Note 1.3- Drag Force**

In investigations pertaining to particle-laden flows within the cardiovascular system, the drag force emerges as a paramount influence. This force opposes the direction of the particle's motion relative to the carrying fluid. It results from the interaction between the particle and surrounding fluid molecules. Factors such as particle shape and surface characteristics influence the magnitude of the drag force. Irregularly shaped or rough-surfaced particles experience higher drag forces. For spherical particles, the magnitude of the drag force is directly linked to the diameter, whereby larger diameters correspond to heightened drag forces. This relationship underscores the significance of particle dimensions in determining the force exerted within the fluid. It plays a significant role in predicting and understanding particle behavior in fluid flow. For spherical particles, the drag force can be calculated by the following equation:

$F_{D}=\left( \frac{1}{\tau_{p}} \right)m_{p}(u-v)$=$\left( \frac{3\eta_{app}C_{D}R_{e_{r}}}{4\rho_{p}d_{p}^{2}} \right)m_{p}\left( u-v \right)$ (S15)

The particle relaxation time $\tau_{p}$, the particle density $\rho_{p}$, and particle diameter $d_{p}$ play essential roles in quantifying the overall drag forces experienced by particles. The drag coefficient, $C_{D}$, the particle relative Reynolds number, denoted as $R_{e_{r}}$is defined $\frac{\rho\left\| \mathbf{u}\boldsymbol{-}\mathbf{v} \right\|d_{p}}{\eta_{app}}$. This number incorporates the fluid's density (ρ), the magnitude of the velocity difference between the particle (u) and the surrounding fluid (v), and the apparent viscosity ($\eta_{app}$) of the fluid [6].

In order to determine the drag coefficient, various standard drag correlations have been employed. These correlations are represented as a piecewise function that depends on the relative Reynolds number. The values and details of these correlations, as reported by Turton and Levenspiel in 1986, are presented in Table 1 [7].

**Supplementary Note 1.4- Particle-particle Interaction Force**

Particle-particle interactions in fluid flows give rise to two distinct types of forces: short-range and long-range. The short-range force operates within extreme proximity, while the long-range force extends to particles separated by a distance of 5-100 nm. The latter force is particularly crucial in understanding particle scattering phenomena.

Researchers often employ the Lennard-Jones potential to characterize the long-range interaction force mathematically. This potential is derived by calculating the gradient of the corresponding potential energy. No studies conducted by [8], [9] have extensively explored the utilization and significance of the Lennard-Jones potential within this context.

| $U\left( r \right)=4\varepsilon\left[ \left( \frac{\sigma}{r} \right)^{12}-\left( \frac{\sigma}{r} \right)^{6} \right]$ | (S16) |
| --- | --- |
| $r_{m}=\sqrt[6]{2} \sigma$ | (S17) |
| $F=-\nabla U$ | (S18) |

where the potential force of each particle, denoted as U, is expressed in terms of the particle radius. The strength of the interparticle interaction is represented by ε, while the impact radius of the particle is denoted as σ.

In calculating the interaction forces between microbubbles, the distance between them is denoted as r, with the minimum value of this distance expressed as $r_{m}$. Equation (19S) is utilized to determine each particle's interaction force. This Equation provides a precise means of calculating the interaction forces, taking into account the parameters mentioned above.

| $F_{p-pi}=\frac{24\varepsilon}{\sigma}\sum_{j=1}^{N} \left[ 2\left( \frac{\sigma}{\left\vert r_{i}-r_{j} \right\vert} \right)^{13}-\left( \frac{\sigma}{\left\vert r_{i}-r_{j} \right\vert} \right)^{7} \right]\left( \frac{r_{i}-r_{j}}{\left\vert r_{i}-r_{j} \right\vert} \right)$ | (S19) |
| --- | --- |
|  |  |

This study sets the cutting length to 55 mm to investigate the particle-particle interaction forces within fluid flows and their influence on particle motion. Previous research conducted by Tan and Liu [10] suggests that when the radius of the rigid particle surface ($r_{m}$), is equal to the particle diameter ($d_{p}$,), specific fixed values are considered for the variables ε and σ. In particular, ε is assigned a value of $\varepsilon={10}^{-15} J$, while σ takes on the value of $\sigma=d_{p}$..

**Supplementary Note 2- Validation**

In research conducted by Kim et al. [11], for different diameters of particles, the path of movement of particles in a Y-shaped tube geometry have been investigated and calculated practically and theoretically. A similar geometry used previously is utilized to validate the movement paths of particles and fluid flow. Dimensions of particles such that their total density of $5230(\frac{kg}{m^{3}})$ is equal to $3, 5$and $7$ micrometers. The viscosity and density of the fluid inside the pipe, which are also governed by the laminar flow regime (with flow rates of $4, 8$, and $12$ liters per minute), are considered $1.78\times{10}^{-5}(Pa.s)$ and $1.78(\frac{kg}{m^{3}})$, respectively. In the simulation section related to this geometry, which can be seen in **Fig. S1a**, the particles are entered randomly from the inlet (1000 particles) and disappear after reaching the outlet section. The walls of this geometry are also conditioned as stick walls. According to **Fig. S1a**, the dimensions of the geometry are as follows:

${\theta={37.5}^{^{\circ}},L}_{1}=10cm$, $L_{2}=5 cm$, $a=0.5 cm, b=0.4 cm$

The flow velocity resulting from the simulation is shown in **Fig. S1a**. Furthermore, the results of particle tracking for times $t=0.005 (s)$and $t=0.5 (s)$ are shown in **Figs. S1b** and **S1c**, respectively. It should be noted that there is a stationary point for particles in the flow at the bifurcation location, which causes the accumulation of many particles. As shown in **Fig. S1c**, the particles entering the flow near the wall and away from the center of the pipe are subjected to hitting the wall and sticking to it (due to the boundary condition assumption). According to **Fig. S1d**, the theoretical results performed in this work for the decomposition rate of particles are in good agreement with the experimental results obtained by Kim et al. [11].

Magnetic forces affect particles and change their movement paths. In other words, as a result of placing a permanent magnet, a magnetic field is created around the particle, which causes a force corresponding to the location of that particle. Suppose the location of the permanent magnet installation is chosen correctly. In that case, the direction of these forces will increase the contact between the drug nanocarriers and the atherosclerosis blockage that causes more efficient drug delivery. Based on the relationship obtained by Camacho and Sosa (Camacho & Sosa, 2013) for a cubic permanent magnet, the magnetic flux density along the central axis of the magnet can be calculated:

| $B\left( y \right)=\frac{\mu_{0}M}{\pi}\left[ arctan\frac{ab}{(y-c)\sqrt{a^{2}+b^{2}+{(y-c)}^{2}}}-arctan\frac{ab}{(y+c)\sqrt{a^{2}+b^{2}+{(y+c)}^{2}}} \right]$ | (S20) |
| --- | --- |

The magnet dimensions are displayed by parameters $a,b$, $c$ and$\mu_{0}M$ indicating magnetization. The magnetic field created by a permanent magnet is considered in the research of Manshadi et al. [12] to validate the calculations of the magnetic field and its related parameters. The dimensions of this magnet are reported in **Fig. S2a**, and $B_{rem}$ is equal to 0.8 T. Then, the magnetic flux density is calculated based on a model similar to the mentioned model. It has been compared with the numerical results of that research [12] and the theoretical relationship of Sosa and Camcho by considering $\mu_{0}M=(0.87+\pm0.07\left( T \right))$. These results are shown in **Fig. S2b** and **S2c,** which show that a good match between the magnetic field of each model. In this way, the correctness of the method used during this research is proven.

**Supplementary Note 3- Computational method and molecular models**

The results of the MD simulation, which is performed based on the structure of the MOF structure, and the density and different diameters, show that the percentage of the drug is according to **Fig .2**. Also, the structure of the ZIF-8, *losartan potassium*, and *ZIF-8 loaded with losartan potassium* , are shown in Fig. S3.

**Supplementary Note 4- Mesh study and Solution Dependence to Time**

In order to model the movement of nanoparticles under a magnetic field, two modules of fluid flow and magnetic field were used in the geometry of the carotid artery. The Mesh fluid flow network with and without a magnetic field has 31290 and 178264 elements, respectively. The simulation result (magnitude of fluid velocity in outlets) shows a difference of less than 3% for 31290 and 62090 elements. In order to ensure the effect of meshing quality on the results, meshing was done with different networks. Due to minimal difference in results and to reduce time and cost, 31290 elements were used.

**Supplementary Note 5- Carrier adhesive dynamics model**

**Fig. S5** Indicates the drug transfer capabilities of five different types of metal-organic frameworks (MOFs) with similar sizes of particles. The MOF-801 significantly influences medication across all diameter ranges among the tested MOFs. At a diameter of 300 nm, MOF-801 exhibits the highest drug transmissibility compared to other MOFs, with an optimized average transferred drug density of 7268 ($\frac{pg}{m^{2}}$). UI-66 performs favorably for diameters above 200 nm, with an average drug transfer density of 7500 ($\frac{pg}{m^{2}}$) for 300 nm and 2200 ($\frac{pg}{m^{2}}$) for 200 nm diameter.

**Supplementary Note 6- fluid flow Characteristics and Magnetic Field Results**

The blood flow pattern inside the carotid artery, which enters the common carotid in the form of a pulse, has been investigated in many numerical and experimental studies [13], [14].In this study, only a part of the results is discussed. The streamline of the blood velocity in the first and second peaks and the lowest are shown in **Fig. S6a**. It can be seen that the magnitude of the inlet velocity strongly influences the maximum value of the flow velocity inside the carotid artery. Also, the flow rate at the bifurcations is higher than in the common carotid area due to the small diameter of the artery. In addition, according to **Fig. S6b**, which shows the blood velocity magnitude in the carotid artery in slice form, it is clear that the blood velocity magnitude increases in the areas of small artery diameter. It is worth mentioning that in the areas of arterial blockage, due to the considerable reduction in the diameter of the artery, the flow velocity value increases dramatically, which has also been observed in previous studies [13], [14].

The amount of magnetic field created inside the carotid artery is essential in determining the orientation of the movement of the drug carrier. In our previous study [2], we optimized the placement of magnets near the carotid artery. In the present study, we have used the Helbach arrangement by placing it before the bifurcation of the carotid near the body skin because allocating the magnet is to direct more NCs to the target branch (the internal carotid artery that has atherosclerosis). The direction and magnitude of the magnetic flux density in the carotid artery are shown in **Fig. S7a**and**S7b**, respectively. It can be seen that the magnitude of the magnetic field near and ahead of the bifurcation has the highest value, and the direction of the field is in the direction where NCs can be drawn toward the inner branch of the carotid artery.

**Supplementary Note 7- Mesh Independency Analysis**

To ensure the authenticity of results and validate the robustness of numerical outcomes, a mesh independence analysis was conducted. For this purpose, three distinct meshes were considered: physics-controlled coarse and normal meshes, comprising 47,237 and 80,978 elements, respectively, and a user-controlled fine mesh with 135,146 elements. Two separate cutlines were established, one in the plaque-affected outlet, connecting points [0.0455, -0.1800, -0.9820] and [0.04562, -0.1762, -0.9804], as depicted in **Fig. S1**. Another cutline was drawn in the unaffected outlet **(Fig. S2)**, aligning with points [0.04081, -0.1864, -0.982] and [0.04563, -0.1762, -0.9804] – all coordinates are in meters. Velocities are computed for all points along these cutlines, and the results, with length nondimensionalization adjustment applied to both cutlines, are depicted in **Fig. S8 and Fig. S9.** Based on the velocity plots for cutlines illustrated in **Fig. S9** and **Fig. S10,** the normal mesh size exhibited remarkable accuracy. The profiles of normal and fine meshes exhibit striking similarity; with minimal differences observed in both their magnitudes and shapes, and the subtle variations between the two curves are negligible. This suggests that increasing the number of elements, or in other words, improving mesh quality, is not necessary and only leads to higher computational costs. Therefore, opting for a normal mesh is a more reasonable choice.

**Supporting Figure Legends**

***Supplementary Figure 1.*** *(a) Drawing a Y-shaped single branch tube congruous to Kim et al.* [11] *studied to validate the fluid flow solution and particle tracking solution. (b) The contours of the fluid’s velocity inside the whole domain with the 8 lit/min flow rate. (c) The motion path of ~*$5 \mu m$ *diameter particle at* $t=0.5 s$*, (d) comparing particle deposition percentage resulting from the current study versus those of the previous studies.*

***Supplementary Figure 2.*** *The studied magnet for the validation of the generated magnetic field surrounding it. (a) A schematic of the 2D magnet. (b) Simulation results correspond to the contours of the magnetic flux density inside a 2D magnet with Brem=0.8 (T) and in the surrounding area. (c) comparing the y component value of the magnetic flux density surrounding the magnet with the results of Manshadi et al.’s study* [12]*, and the theoretical analysis done by Camacho and Sosa.*

***Supplementary Figure 3.*** *(A)* *Two-dimensional view (B) Three-dimensional view of the unit cell of ZIF-8 (C) losartan potassium three- structure (D), losartan potassium two- structure (E) ZIF-8 loaded with losartan potassium*

***Supplementary Figure 4.*** *Transmission probability of nanocarriers to the internal carotid for different sizes of nanocarriers (A) PCN-224 nanocarrier, (B) ZIF-90 nanocarrier, (C) MOF-801 nanocarrier, (D) ZIF-8 nanocarrier, (E) UIO-66 nanocarrier.*

***Supplementary Figure 5.*** *Comparison between statistical analysis (t-test) on different MOF types in a wide range of diameters in four Nanocarrier injections*

***Supplementary Figure 6.*** *In the carotid artery domain simulation, streamlining of velocity and pressure in a cardiac cycle in the blood artery are described at three different times, (a) the first velocity peak at time t=2.32 s, (b) the minimum velocity at time t=2.51 s, and (c) the second velocity peak at time t=2.62 s are considered. (d) Display of velocity surface sections in carotid geometry at the second velocity peak.*

***Supplementary Figure 7.*** *Magnetic field distribution and its effect on carotid artery amplitude (a) The magnitude of magnetic flux density under the external arrangement of a Helbach magnet. (b) The magnitude and direction of the magnetic flux density in the geometry of the carotid artery under the influence of the external magnet.*

***Supplementary Figure 8.*** *In the carotid artery domain simulation, NCs dispersion in the carotid artery domain for (a) the first velocity peak at time t=2.32 s and (b) the minimum velocity at time t=2.51 s. (C)* *The path line of NCs inside the carotid artery domain.*

**Supplementary Figure 9.** The velocity magnitudes along the cutline of plaque-afflicted carotid outlet, as assessed for coarse, normal, and fine meshes, indicates that a normal mesh would meet the precision requirements for this study. The differences in velocity profiles between normal and fine meshes, despite the computational cost associated with fine meshing, are negligible. x* represents non-dimensional length of cutline A-A.

**Supplementary Figure 10.** The computed velocity magnitudes along the depicted cutline of the unaffected carotid outlet further underscores the appropriateness of selecting a normal mesh for this study. Despite a substantial difference in the number of elements between normal and fine meshes, their results are remarkably similar. Here, x* denotes the non-dimensional length along cutline B-B.

***Supplemental Figures***

***Supplementary Figure 1***


***Supplementary Figure* 2**

***Supplementary Figure* 3**

| 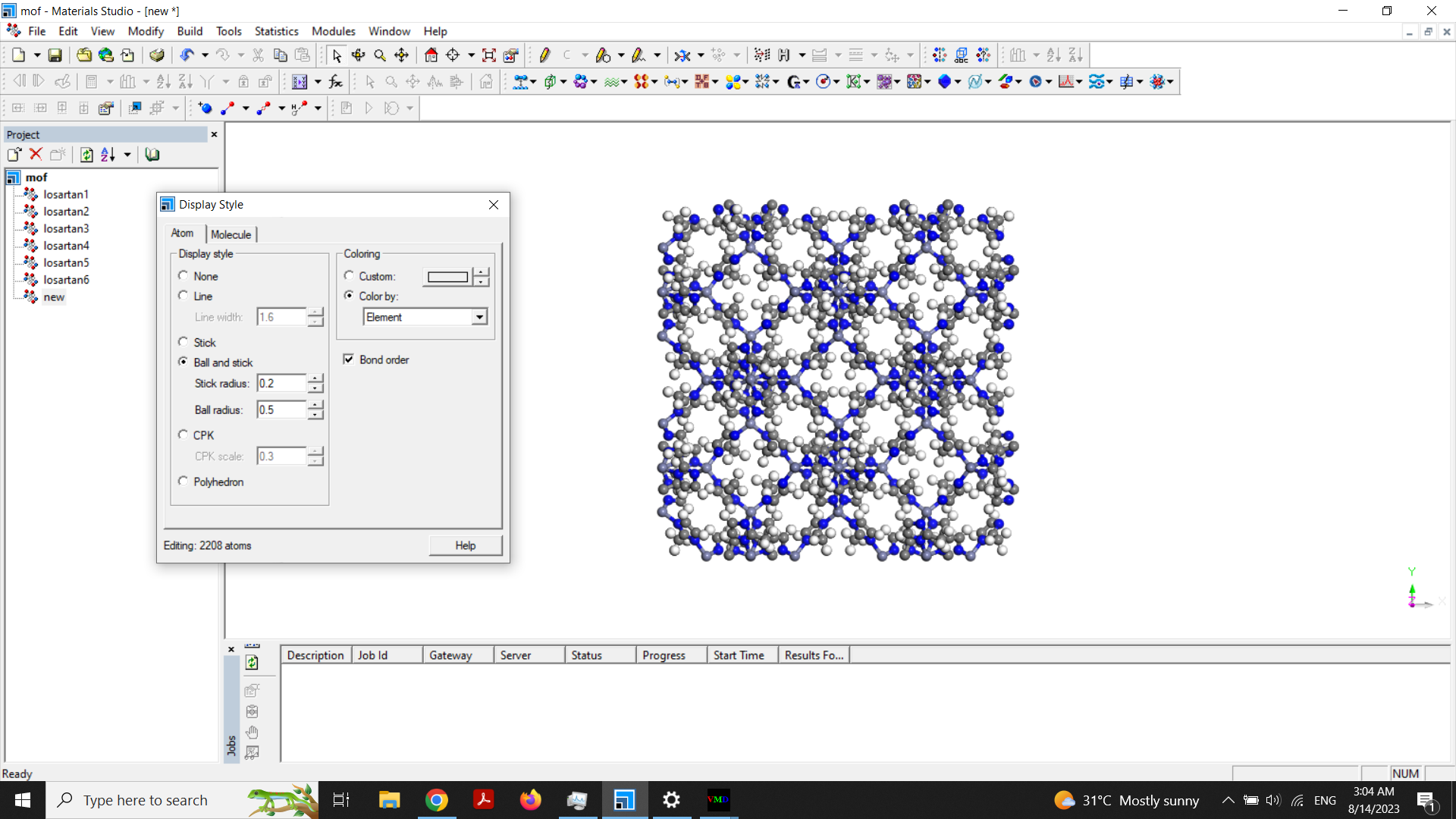  **(A)** | 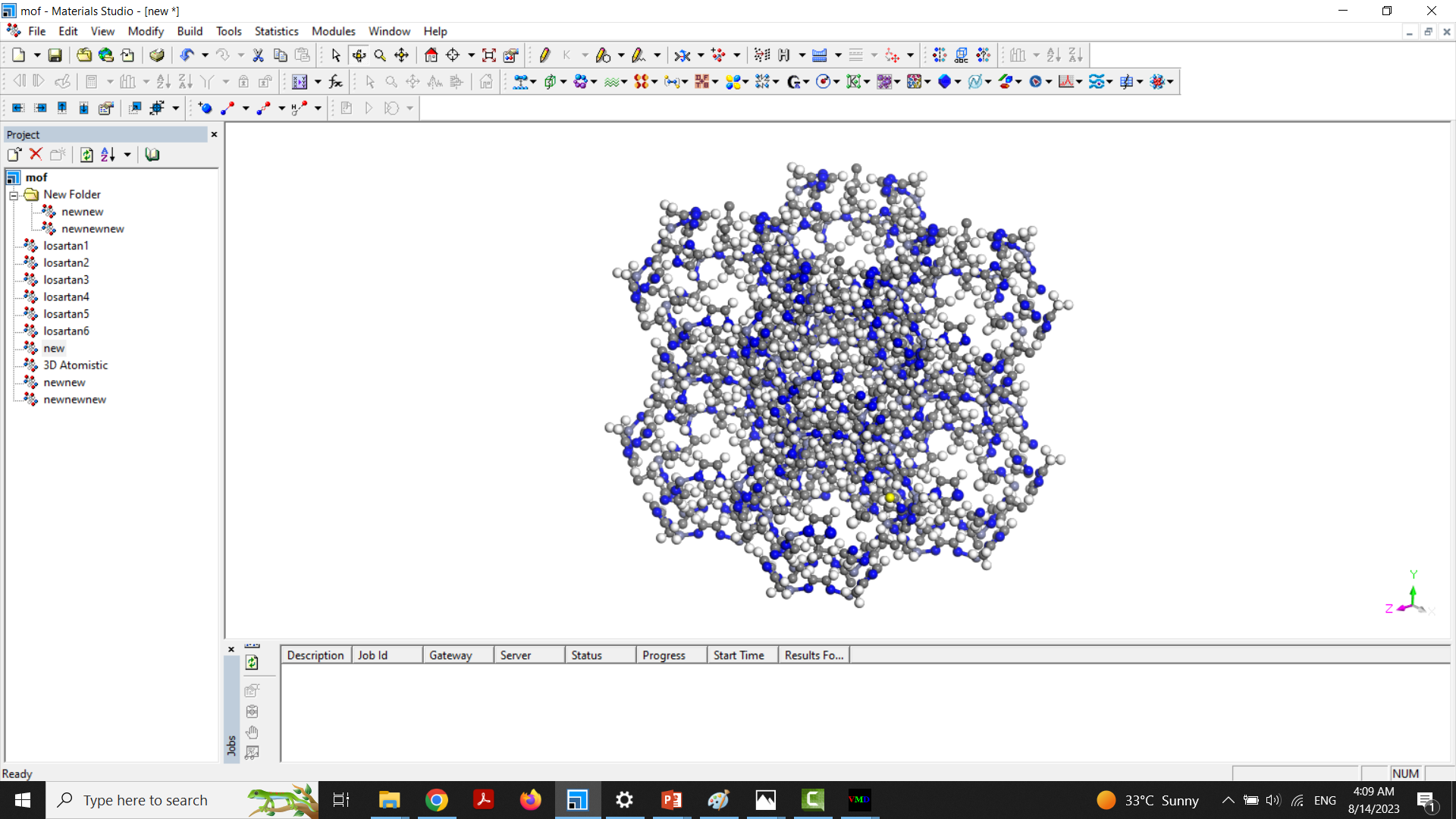  **(B)** |
| --- | --- |
| 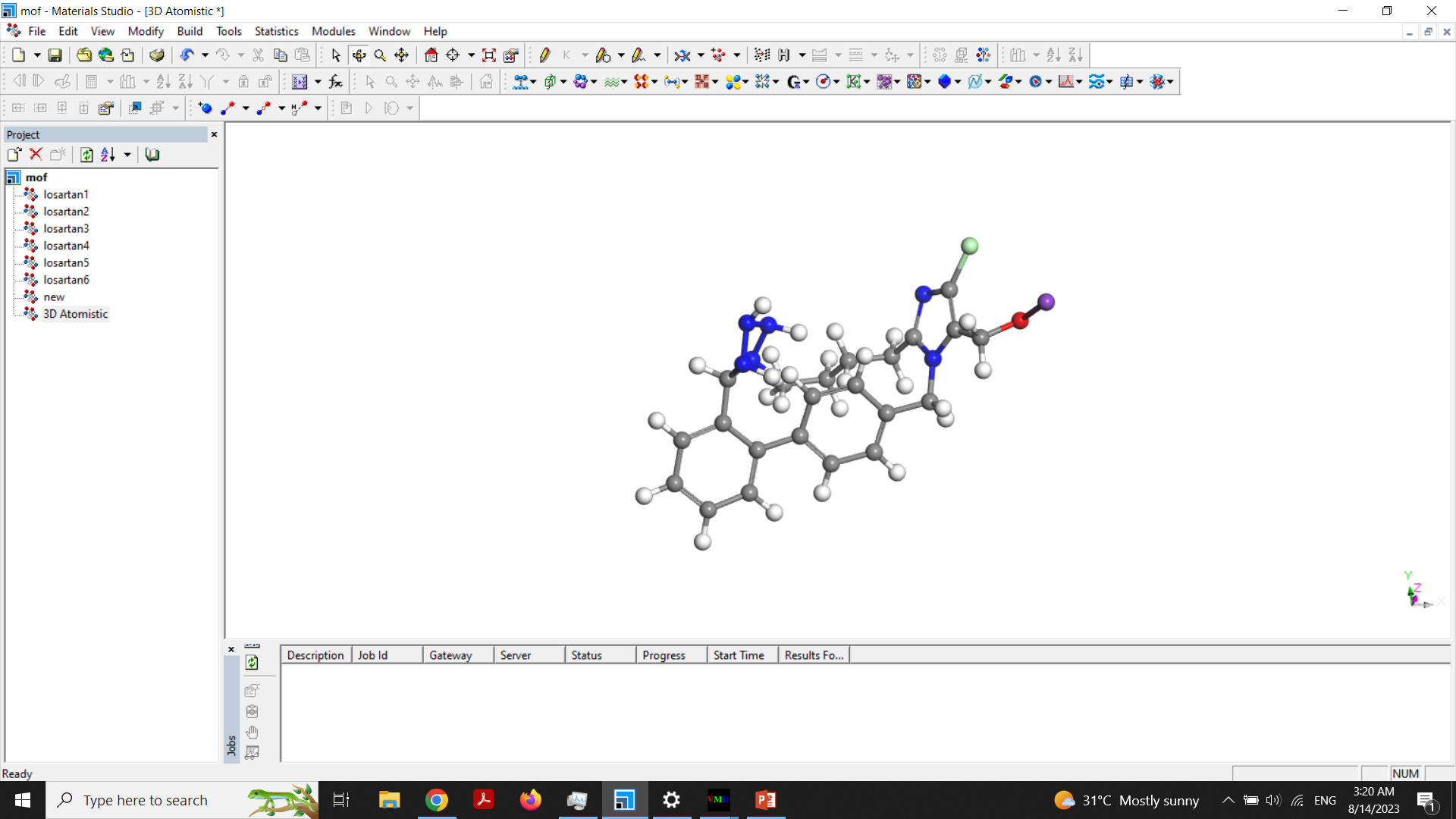  **(C)** | **(D)**  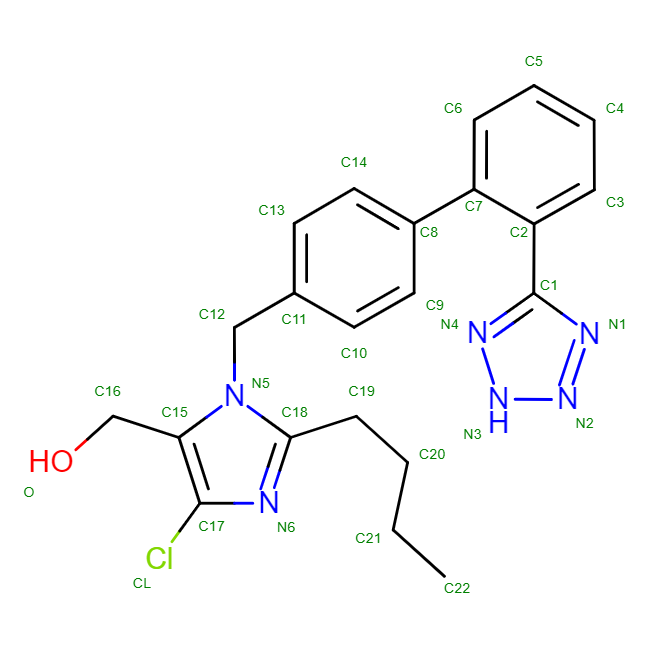  P |
| 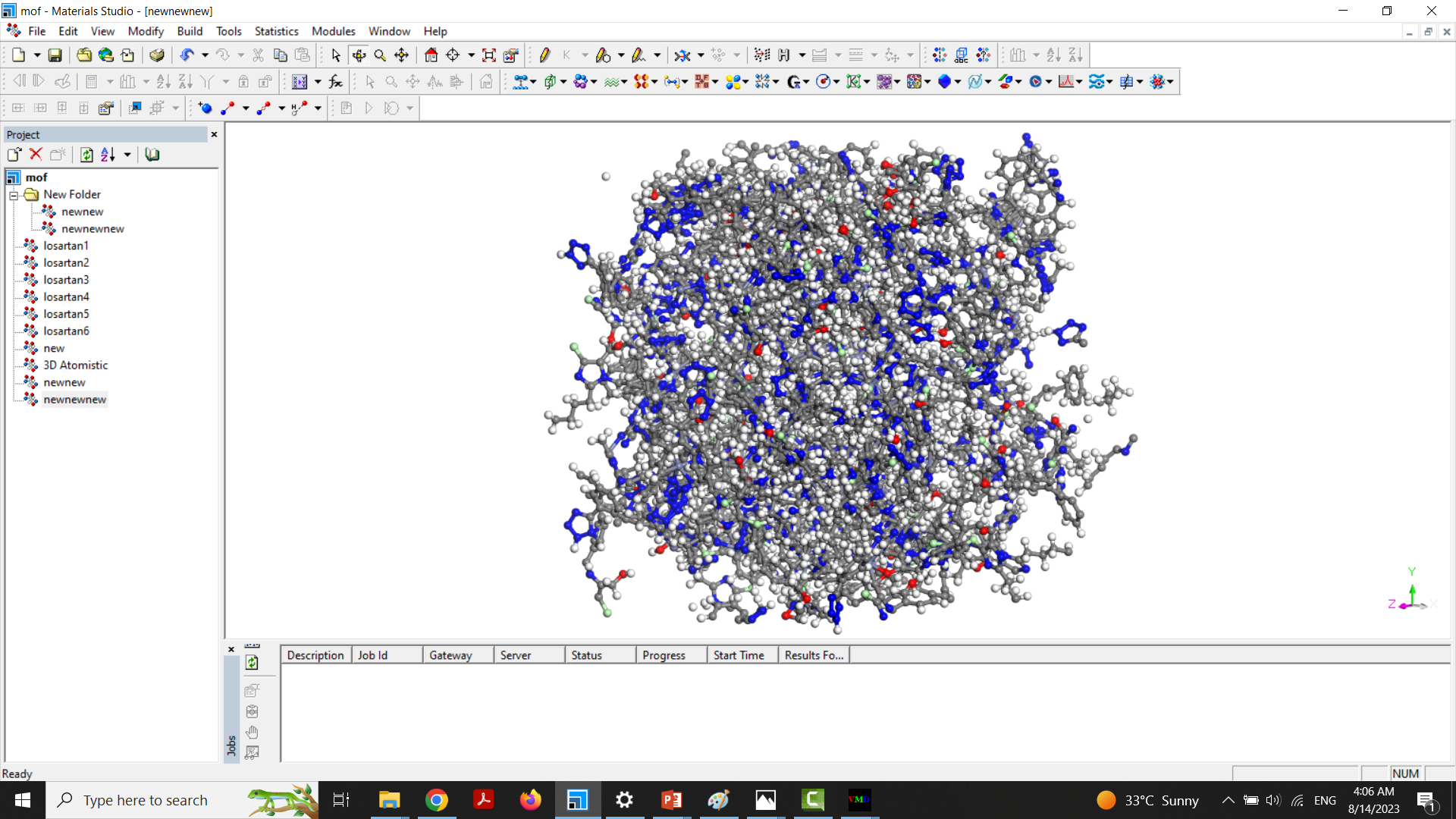  **(E)** | |

***Supplementary Figure* 4**

**
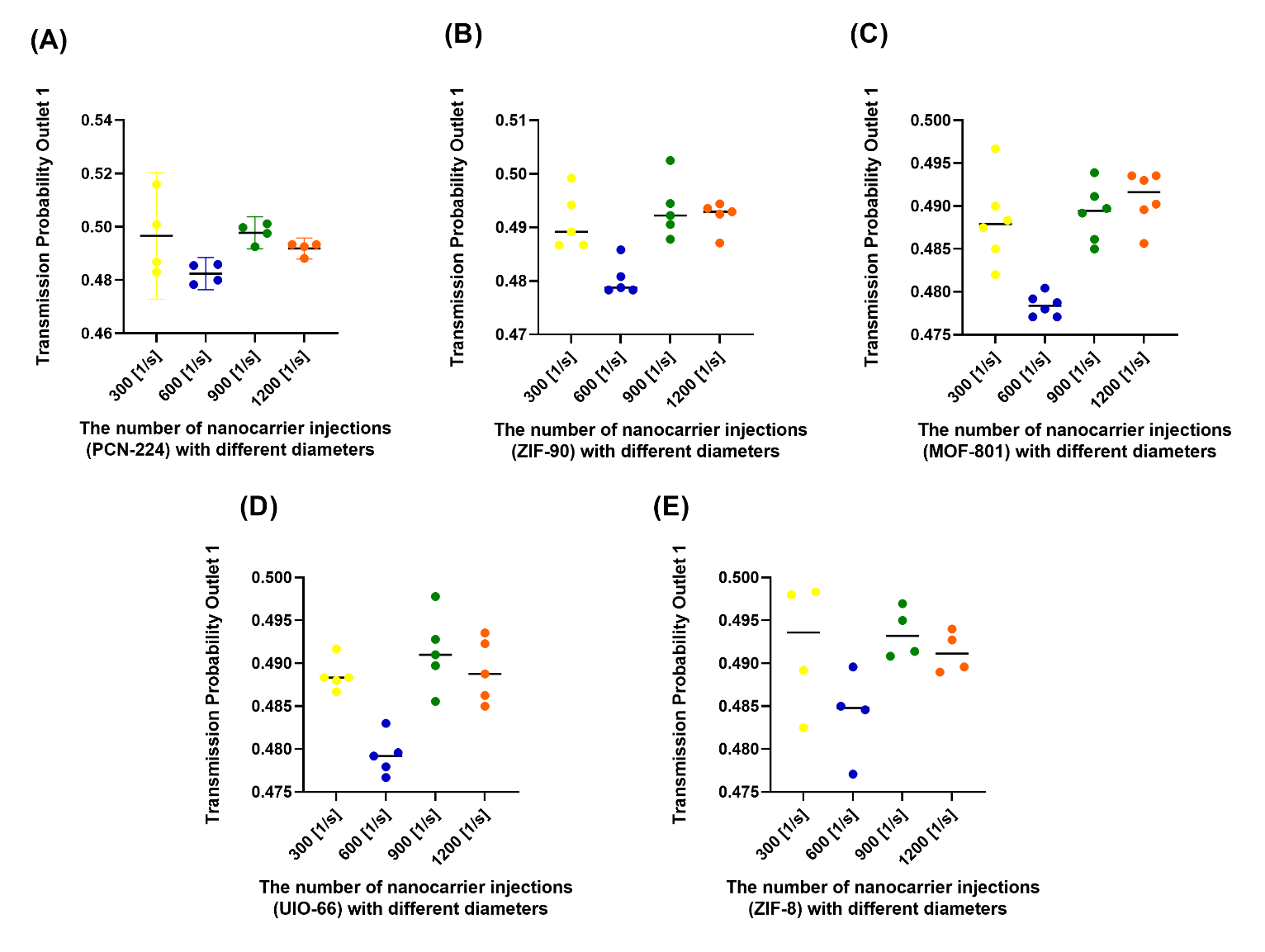
*Supplementary Figure* 5**


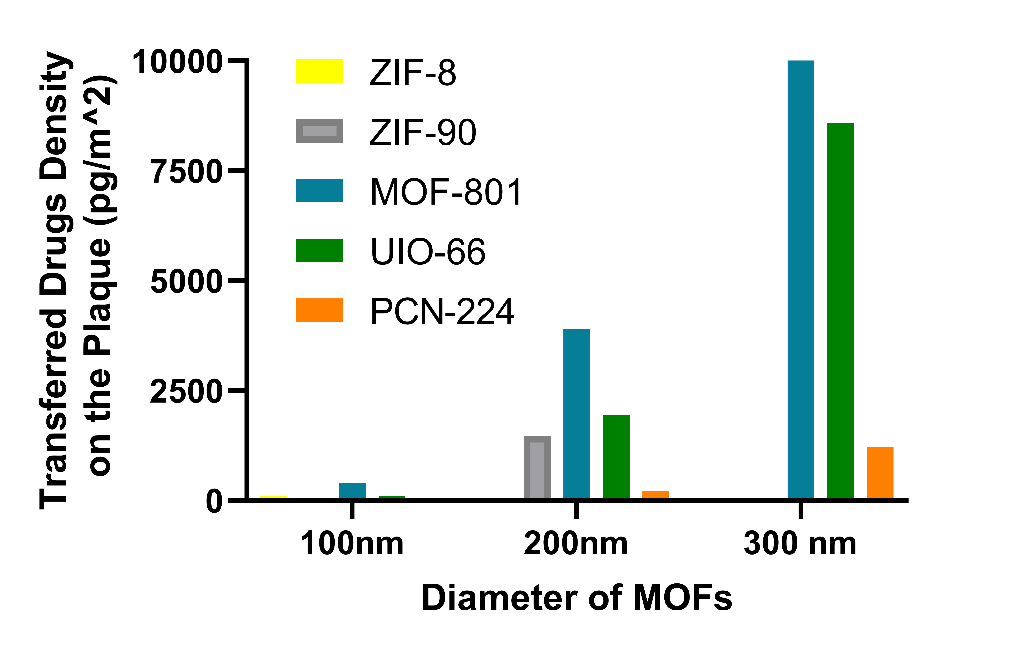


***Supplementary Figure* 6**

***Supplementary Figure* 7**


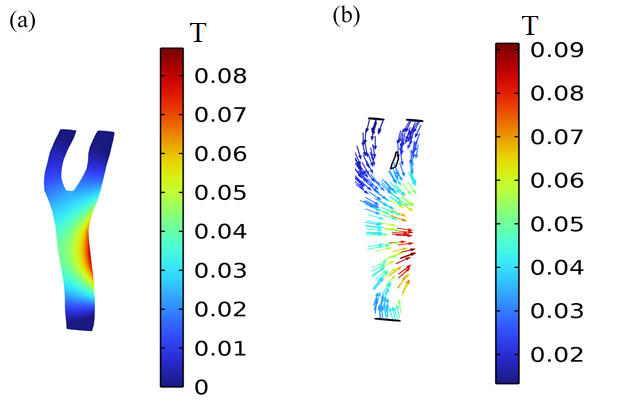
***Supplementary Figure* 8**

***Supplementary Figure* 9**


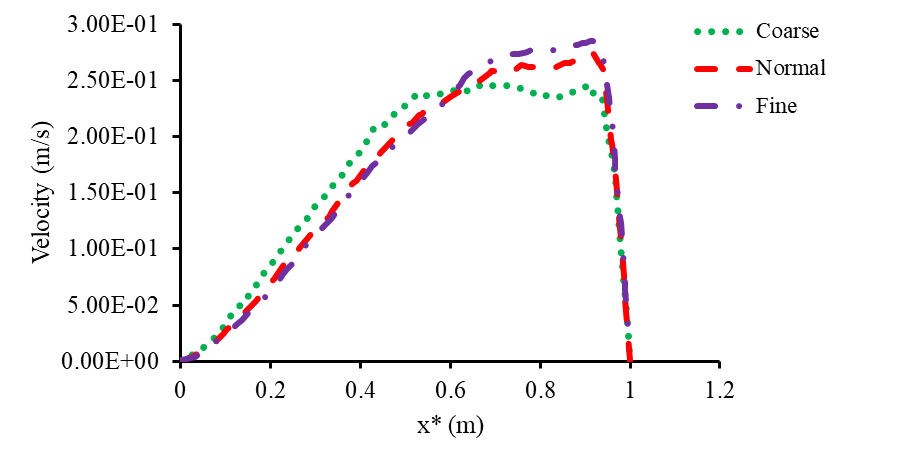

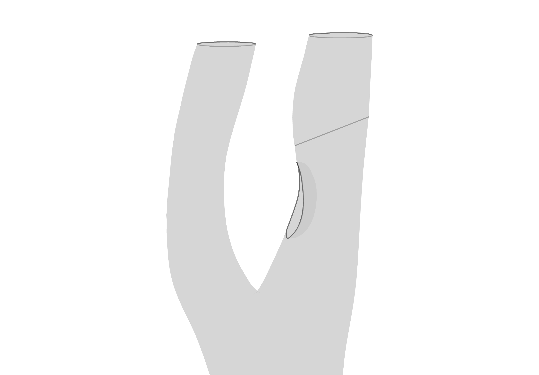


z

y

**A**

**A**

**A**

**A**

***Supplementary Figure 10***


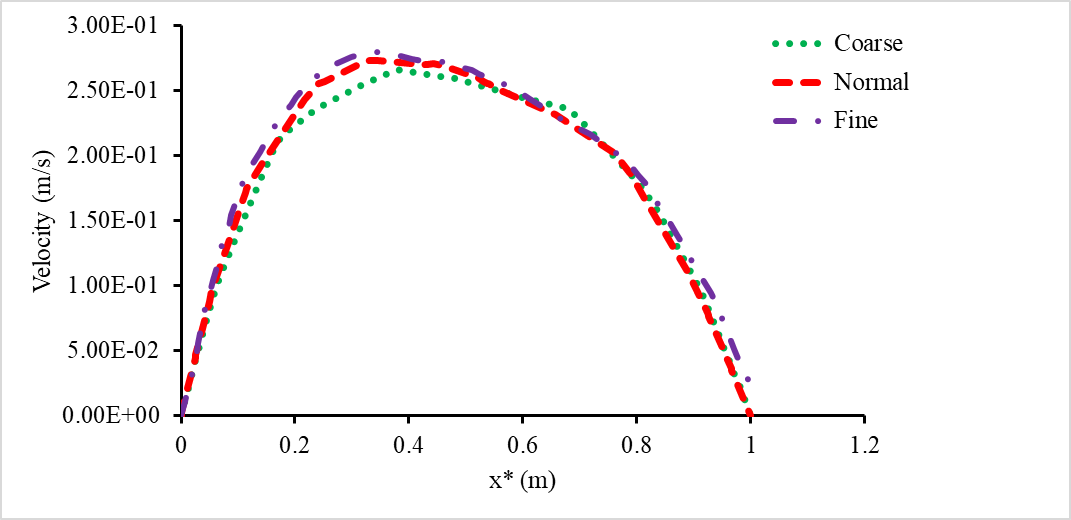

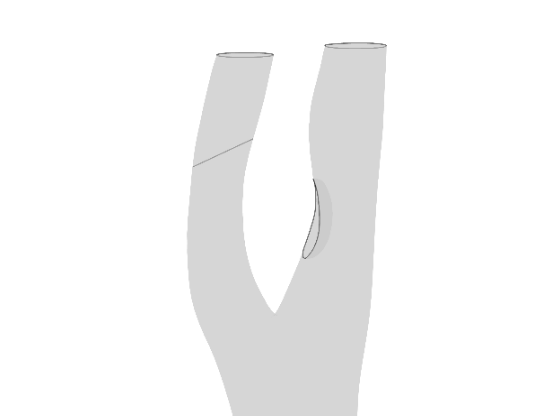


z

y

**B**

**B**

***Tables***

***Supplementary Table 1.*** The drag coefficient values for various ranges of *Re_r._*

Range Correlation

${Re}_{r}$ $\leq0.01$ $C_{D}=\frac{24}{{Re}_{r}}$(1 + $\frac{3}{16}{Re}_{r})$

$0.1\leq{Re}_{r}$ $\leq20$ $C_{D}=\frac{24}{{Re}_{r}}$(1 + 0.1315${{Re}_{r}}^{(0.82-0.05 log{Re}_{r})}$)

$20\leq{Re}_{r}$ $\leq260$ $C_{D}=\frac{24}{{Re}_{r}}$(1 + 0.1935${{Re}_{r}}^{0.6305}$)

$260\leq{Re}_{r}$ $\leq1500$ log $C_{D}=1.6435-1.1242 log{Re}_{r}+(0.1558 {(log{Re}_{r})}^{2}$

***Supplementary Table 2:*** *Parameter of fixed pressure and energy*

| Equilibration steps | $1000000$ |
| --- | --- |
| Production steps | $1000000$ |
| Force field | Universal |
| Charges | Charge using QEq |
| Van der Waals terms: summation | Atom based |
| Sample interval | $50$ steps |
| Spline width | $1A$ |
| Buffer width | $0.5A$ |
| Cutoff distance | $12.5A$ |
| Truncation method | Cubic spline |
| Electrostatic terms: summation method | Ewald |
| Buffer width | $0.0001 Kcal/mol$ |
| Accuracy | $0.5A$ |

| Ensemble | $NVT$ |
| --- | --- |
| Temperature | $310$ |
| Duration | $100Ps$ |
| Q ratio | $0.10000$ |
| Number of steps | $100000$ |
| Initial velocities | Random |
| Control method | Nose |
| Force field | Universal |
| Charges | use current |
| Van der Waals terms: summation method | Atom based |
| Sample interval | $50$ steps |
| Spline width | $1A$ |
| Buffer width | $0.5A$ |
| Cutoff distance | $13.5A$ |
| Truncation method | Cubic spline |
| Electrostatic terms: summation method | Ewald |
| Buffer width | $0.0001 Kcal/mol$ |
| Accuracy | $0.5A$ |
|  |  |

***Supplementary Table 3 :****Parameter of energy and dynamics for MD simulation*

.

| **Parameter** | **Value** |
| --- | --- |
| $m_{r}$ | ${10}^{14} 1/m^{2}$ |
| $\lambda$ | ${10}^{-10} m$ |
| $k_{B}$ | $1.38\times{10}^{-23}m^{2}Kg/s^{2}K$ |
| $T$ | $300 K$ |
| $\delta_{\mathrm{eq}}$ | $5\times{10}^{-9}$ |
|  |  |

***Supplementary Table 4:*** Constants required for calculating the adhesion probability of microcarriers [15].

***Supplementary Table 5:*** *Five types of MOFs are investigated in the current study. The size,*

*density, wt%, and three-structure are presented.*

| **MOF** | **Diameter (nm)** | **Density (kg/**$\boldsymbol{m}^{\boldsymbol{3}}\boldsymbol{)}$ | **Three-structure** |
| --- | --- | --- | --- |
| ZIF-8 | 78.8  105.7  130  150  [1, 2] | 950[3] | 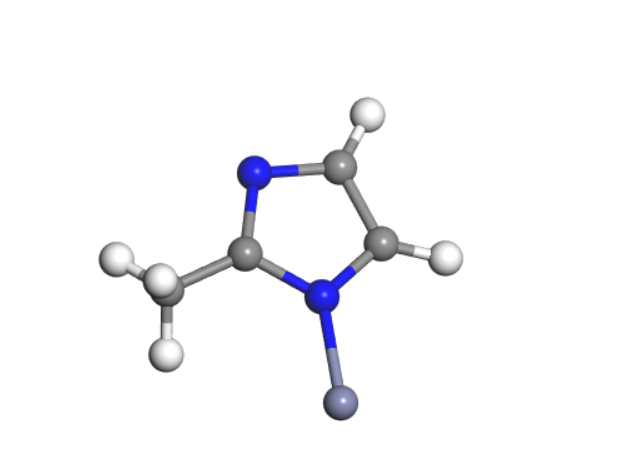 |
| ZIF-90 | 70  90  120  150  200  [4, 5] | 1110  [6] | 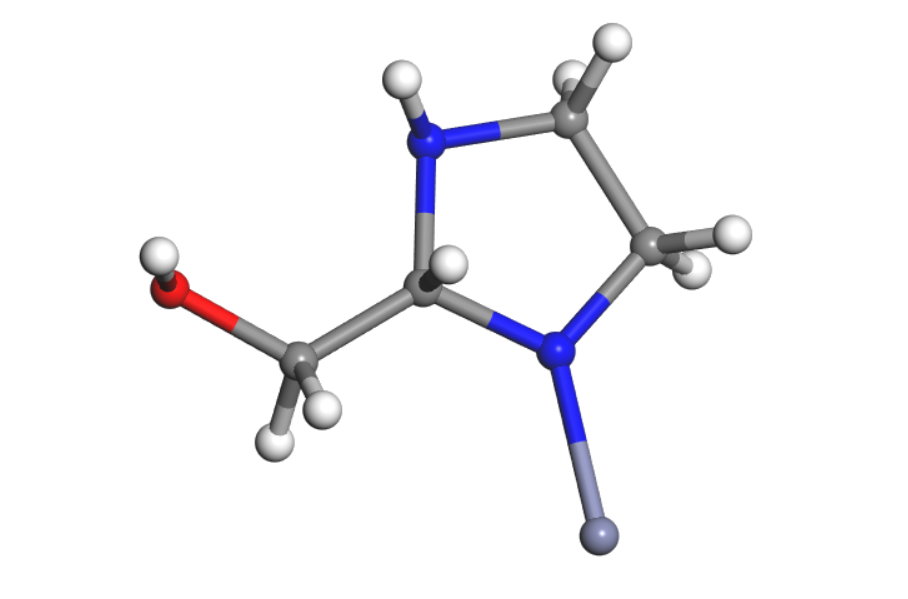 |
| PCN-224 | 90  150  180  300  [7, 8] | 508  [9] | 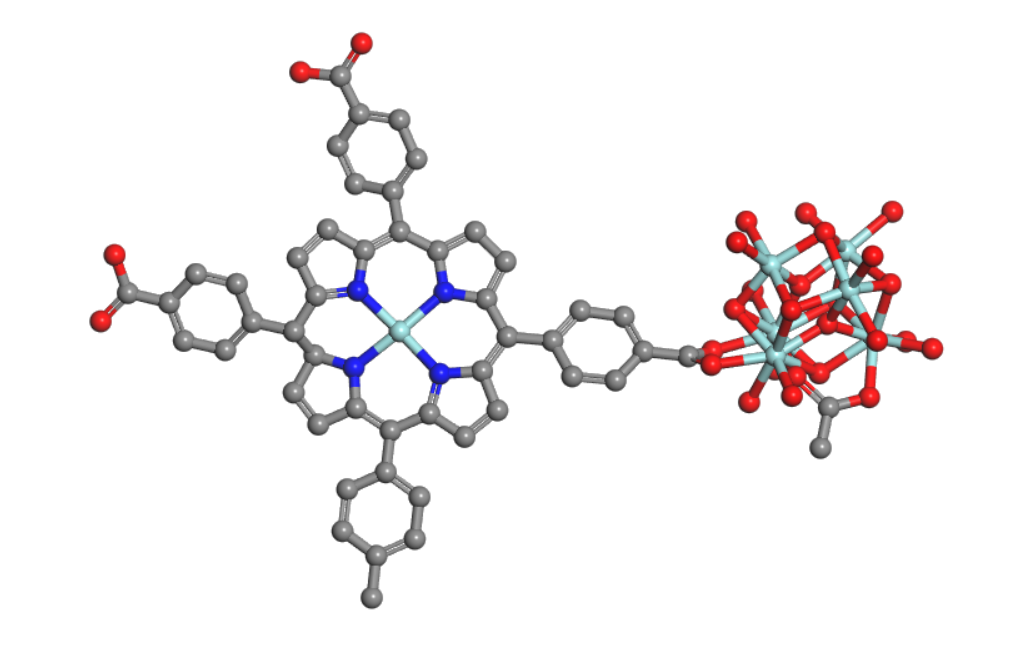 |
| MOF-801(Zr) | 100  150  200  250  300  [10, 11] | 1592  [12] | 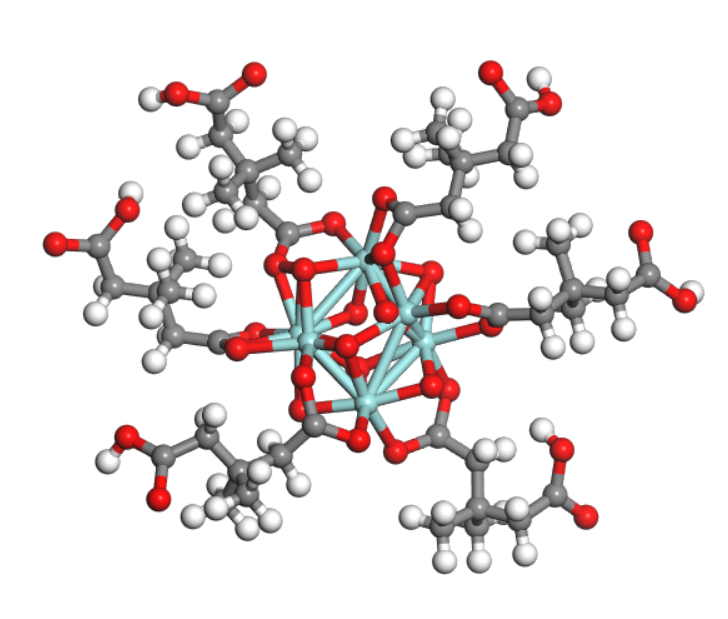 |
| UIO-66 | 72  140  208  270  319  150  319  [13, 14] | 1237  [15] | 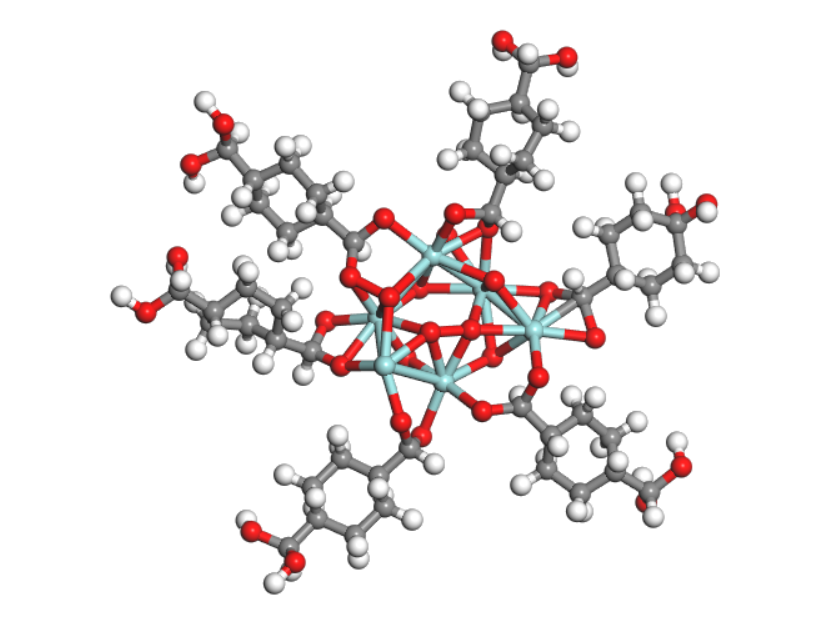 |

***Supplementary Table 6:*** ***T-test analysis related to UIO-66***

| UIO-66 | 300 [1/s] | 600 [1/s] | 900 [1/s] | 1200 [1/s] |
| --- | --- | --- | --- | --- |
| Theoretical mean | 0/000 | 0/000 | 0/000 | 0/000 |
| Actual mean | 0/4886 | 0/4793 | 0/4914 | 0/4892 |
| Number of values | 5 | 5 | 5 | 5 |
|  |  |  |  |  |
| One sample t test |  |  |  |  |
| t, df | t=591/3, df=4 | t=450/8, df=4 | t=246/2, df=4 | t=295/2, df=4 |
| P value (two tailed) | <0/0001 | <0/0001 | <0/0001 | <0/0001 |
| P value summary | **** | **** | **** | **** |
| Significant (alpha=0.05)? | Yes | Yes | Yes | Yes |
|  |  |  |  |  |
| How big is the discrepancy? |  |  |  |  |
| Discrepancy | 0/4886 | 0/4793 | 0/4914 | 0/4892 |
| SD of discrepancy | 0/001848 | 0/002377 | 0/004464 | 0/003705 |
| SEM of discrepancy | 0/0008263 | 0/001063 | 0/001996 | 0/001657 |
| 95% confidence interval | 0/4863 to 0/4909 | 0/4763 to 0/4822 | 0/4858 to 0/4969 | 0/4846 to 0/4938 |

***Supplementary Table 7:*** ***T-test analysis related to PCN-224***

| PCN-224 | 300 [1/s] | 600 [1/s] | 900 [1/s] | 1200 [1/s] |
| --- | --- | --- | --- | --- |
| Theoretical mean | 0/000 | 0/000 | 0/000 | 0/000 |
| Actual mean | 0/4966 | 0/4824 | 0/4977 | 0/4918 |
| Number of values | 4 | 4 | 4 | 4 |
|  |  |  |  |  |
| One sample t test |  |  |  |  |
| t, df | t=66/40, df=3 | t=254/2, df=3 | t=263/6, df=3 | t=394/6, df=3 |
| P value (two tailed) | <0/0001 | <0/0001 | <0/0001 | <0/0001 |
| P value summary | **** | **** | **** | **** |
| Significant (alpha=0.05)? | Yes | Yes | Yes | Yes |
|  |  |  |  |  |
| How big is the discrepancy? |  |  |  |  |
| Discrepancy | 0/4966 | 0/4824 | 0/4977 | 0/4918 |
| SD of discrepancy | 0/01496 | 0/003795 | 0/003777 | 0/002493 |
| SEM of discrepancy | 0/007479 | 0/001898 | 0/001888 | 0/001246 |
| 95% confidence interval | 0/4728 to 0/5204 | 0/4764 to 0/4884 | 0/4917 to 0/5037 | 0/4879 to 0/4958 |
| R squared (partial eta squared) | 0/9993 | 1/000 | 1/000 | 1/000 |

***Supplementary Table 8:*** ***T-test analysis related to ZIF-90***

| ZIF-90 | 300 [1/s] | 600 [1/s] | 900 [1/s] | 1200 [1/s] |
| --- | --- | --- | --- | --- |
| Theoretical mean | 0/000 | 0/000 | 0/000 | 0/000 |
| Actual mean | 0/4912 | 0/4804 | 0/4935 | 0/4921 |
| Number of values | 5 | 5 | 5 | 5 |
|  |  |  |  |  |
| One sample t test |  |  |  |  |
| t, df | t=202/6, df=4 | t=335/7, df=4 | t=197/5, df=4 | t=381/3, df=4 |
| P value (two tailed) | <0/0001 | <0/0001 | <0/0001 | <0/0001 |
| P value summary | **** | **** | **** | **** |
| Significant (alpha=0.05)? | Yes | Yes | Yes | Yes |
|  |  |  |  |  |
| How big is the discrepancy? |  |  |  |  |
| Discrepancy | 0/4912 | 0/4804 | 0/4935 | 0/4921 |
| SD of discrepancy | 0/005420 | 0/003200 | 0/005588 | 0/002885 |
| SEM of discrepancy | 0/002424 | 0/001431 | 0/002499 | 0/001290 |
| 95% confidence interval | 0/4844 to 0/4979 | 0/4764 to 0/4844 | 0/4866 to 0/5004 | 0/4885 to 0/4957 |

***Supplementary Table 9:*** ***T-test analysis related to MOF-801***

| MOF-801 | 300 [1/s] | 600 [1/s] | 900 [1/s] | 1200 [1/s] |
| --- | --- | --- | --- | --- |
| Theoretical mean | 0/000 | 0/000 | 0/000 | 0/000 |
| Actual mean | 0/4883 | 0/4784 | 0/4892 | 0/4909 |
| Number of values | 6 | 6 | 6 | 6 |
|  |  |  |  |  |
| One sample t test |  |  |  |  |
| t, df | t=240/0, df=5 | t=902/0, df=5 | t=367/7, df=5 | t=386/7, df=5 |
| P value (two tailed) | <0/0001 | <0/0001 | <0/0001 | <0/0001 |
| P value summary | **** | **** | **** | **** |
| Significant (alpha=0.05)? | Yes | Yes | Yes | Yes |
|  |  |  |  |  |
| How big is the discrepancy? |  |  |  |  |
| Discrepancy | 0/4883 | 0/4784 | 0/4892 | 0/4909 |
| SD of discrepancy | 0/004983 | 0/001299 | 0/003259 | 0/003109 |
| SEM of discrepancy | 0/002034 | 0/0005304 | 0/001330 | 0/001269 |
| 95% confidence interval | 0/4830 to 0/4935 | 0/4771 to 0/4798 | 0/4857 to 0/4926 | 0/4877 to 0/4942 |

***Supplementary Table 10:*** ***T-test analysis related to ZIF-8***

| ZIF-8 | 300 [1/s] | 600 [1/s] | 900 [1/s] | 1200 [1/s] |
| --- | --- | --- | --- | --- |
| Theoretical mean | 0/000 | 0/000 | 0/000 | 0/000 |
| Actual mean | 0/4920 | 0/4841 | 0/4935 | 0/4913 |
| Number of values | 4 | 4 | 4 | 4 |
|  |  |  |  |  |
| One sample t test |  |  |  |  |
| t, df | t=129/1, df=3 | t=187/1, df=3 | t=337/5, df=3 | t=404/4, df=3 |
| P value (two tailed) | <0/0001 | <0/0001 | <0/0001 | <0/0001 |
| P value summary | **** | **** | **** | **** |
| Significant (alpha=0.05)? | Yes | Yes | Yes | Yes |
|  |  |  |  |  |
| How big is the discrepancy? |  |  |  |  |
| Discrepancy | 0/4920 | 0/4841 | 0/4935 | 0/4913 |
| SD of discrepancy | 0/007623 | 0/005175 | 0/002925 | 0/002430 |
| SEM of discrepancy | 0/003811 | 0/002588 | 0/001462 | 0/001215 |
| 95% confidence interval | 0/4799 to 0/5041 | 0/4758 to 0/4923 | 0/4889 to 0/4982 | 0/4874 to 0/4952 |

**References**

[1] J. Sheng *et al.*, "Targeted therapy of atherosclerosis by zeolitic imidazolate framework-8 nanoparticles loaded with losartan potassium via simultaneous lipid-scavenging and anti-inflammation," *Journal of Materials Chemistry B,* vol. 10, no. 31, pp. 5925-5937, 2022.

[2] J. C. Tan, T. D. Bennett, and A. K. Cheetham, "Chemical structure, network topology, and porosity effects on the mechanical properties of Zeolitic Imidazolate Frameworks," *Proceedings of the National Academy of Sciences,* vol. 107, no. 22, pp. 9938-9943, 2010.

[3] X. Dou, M. Keywanlu, R. Tayebee, and B. Mahdavi, "Simulation of adsorption and release of doxepin onto ZIF-8 including in vitro cellular toxicity and viability," *Journal of Molecular Liquids,* vol. 329, p. 115557, 2021.

[4] C. G. Jones *et al.*, "Versatile synthesis and fluorescent labeling of ZIF-90 nanoparticles for biomedical applications," *ACS Applied Materials & Interfaces,* vol. 8, no. 12, pp. 7623-7630, 2016.

[5] T. Ge *et al.*, "A bone-targeting drug delivery vehicle of a metal–organic framework conjugate with zoledronate combined with photothermal therapy for tumor inhibition in cancer bone metastasis," *Biomaterials Science,* vol. 10, no. 7, pp. 1831-1843, 2022.

[6] L. Mengwen *et al.*, "The selective and sensitive detection of formaldehyde by ZIF-90-LW via aza-Cope rearrangement," *Analytical Methods,* vol. 12, no. 29, pp. 3748-3755, 2020.

[7] F. Hao, Z.-Y. Yan, and X.-P. Yan, "Size-and shape-dependent cytotoxicity of nano-sized Zr-based porphyrinic metal-organic frameworks to macrophages," *Science of The Total Environment,* vol. 833, p. 155309, 2022.

[8] Y. Zong, S. Ma, J. Gao, M. Xu, J. Xue, and M. Wang, "Synthesis of porphyrin Zr-MOFs for the adsorption and photodegradation of antibiotics under visible light," *Acs Omega,* vol. 6, no. 27, pp. 17228-17238, 2021.

[9] D. Z. Zee and T. D. Harris, "Enhancing catalytic alkane hydroxylation by tuning the outer coordination sphere in a heme-containing metal–organic framework," *Chemical Science,* vol. 11, no. 21, pp. 5447-5452, 2020.

[10] M. Parsaei and K. Akhbari, "MOF-801 as a nanoporous water-based carrier system for in situ encapsulation and sustained release of 5-FU for effective cancer therapy," *Inorganic Chemistry,* vol. 61, no. 15, pp. 5912-5925, 2022.

[11] W.-C. Yun, M.-T. Yang, and K.-Y. A. Lin, "Water-born zirconium-based metal organic frameworks as green and effective catalysts for catalytic transfer hydrogenation of levulinic acid to γ-valerolactone: critical roles of modulators," *Journal of colloid and interface science,* vol. 543, pp. 52-63, 2019.

[12] H. Furukawa *et al.*, "Water adsorption in porous metal–organic frameworks and related materials," *Journal of the American Chemical Society,* vol. 136, no. 11, pp. 4369-4381, 2014.

[13] B. M. Connolly *et al.*, "Tuning porosity in macroscopic monolithic metal-organic frameworks for exceptional natural gas storage," *Nature communications,* vol. 10, no. 1, pp. 1-11, 2019.

[14] W. Morris *et al.*, "Role of modulators in controlling the colloidal stability and polydispersity of the UiO-66 metal–organic framework," *ACS applied materials & interfaces,* vol. 9, no. 39, pp. 33413-33418, 2017.

[15] S. Øien *et al.*, "Detailed structure analysis of atomic positions and defects in zirconium metal–organic frameworks," *Crystal Growth & Design,* vol. 14, no. 11, pp. 5370-5372, 2014.
